# Transcriptome profiling at the transition to the reproductive stage uncovers stage and tissue-specific genes in wheat

**DOI:** 10.1186/s12870-022-03986-y

**Published:** 2023-01-12

**Authors:** Salma Benaouda, Tyll Stöcker, Heiko Schoof, Jens Léon, Agim Ballvora

**Affiliations:** 1 Institute for Crop Science and Resource Conservation, Chair of Plant Breeding, University of Bonn, Bonn, Germany; 2 Institute for Crop Science and Resource Conservation, Chair of Crop Bioinformatics, University of Bonn, Bonn, Germany

**Keywords:** Transition, Shoot apex meristem, Leaves, Double ridge, Reproductive phase, QTL, Transcriptome, Wheat, Flowering time

## Abstract

**Background:**

The transition from vegetative to floral phase is the result of complex crosstalk of exogenous and endogenous floral integrators. This critical physiological event is the response to environmental interaction, which causes biochemical cascades of reactions at different internal tissues, organs, and releases signals that make the plant moves from vegetative status to a reproductive phase. This network controlling flowering time is not deciphered largely in bread wheat. In this study, a comparative transcriptome analysis at a transition time in combination with genetic mapping was used to identify responsible genes in a stage and tissue-specific manner. For this reason, two winter cultivars that have been bred in Germany showing contrasting and stable heading time in different environments were selected for the analysis.

**Results:**

In total, 670 and 1075 differentially expressed genes in the shoot apical meristem and leaf tissue, respectively, could be identified in 23 QTL intervals for the heading date. In the transition apex, Histone methylation *H3-K36* and regulation of circadian rhythm are both controlled by the same homoeolog genes mapped in QTL TaHd112, TaHd124, and TaHd137. *TaAGL14* gene that identifies the floral meristem was mapped in TaHd054 in the double ridge. In the same stage, the homoeolog located on chromosome 7D of *FLOWERING TIME LOCUS T* mapped on chr 7B, which evolved an antagonist function and acts as a flowering repressor was uncovered. The wheat orthologue of transcription factor *ASYMMETRIC LEAVES* 1 (*AS1*) was identified in the late reproductive stage and was mapped in TaHd102, which is strongly associated with heading date. Deletion of eight nucleotides in the *AS1* promoter could be identified in the binding site of the *SUPPRESSOR OF CONSTANS OVEREXPRESSION 1 (SOC1)* gene in the late flowering cultivar*.* Both proteins *AS1* and *SOC1* are inducing flowering time in response to gibberellin biosynthesis.

**Conclusion:**

The global transcriptomic at the transition phase uncovered stage and tissue-specific genes mapped in QTL of heading date in winter wheat. In response to Gibberellin signaling, wheat orthologous transcription factor *AS1* is expressed in the late reproductive phase of the floral transition. The locus harboring this gene is the strongest QTL associated with the heading date trait in the German cultivars. Consequently, we conclude that this is another indication of the Gibberellin biosynthesis as the mechanism behind the heading variation in wheat.

**Supplementary Information:**

The online version contains supplementary material available at 10.1186/s12870-022-03986-y.

## Introduction

A precise adjustment of flowering time to suitable environmental conditions is a critical agronomical factor for successful reproduction [1]. This adaptive trait of transition from vegetative to the reproductive stage is controlled genetically by monitoring and responding to specific seasonal stimuli such as temperature and photoperiod with additional involvement of nutrient availability. Most of the knowledge and understanding of flowering time regulation is gained from the diploid model dicotyledonous plant *Arabidopsis.* Floral transition in *Arabidopsis* implicates six known pathways: age, vernalization, Gibberellin (GA), ambient temperature, photoperiod-dependent, and autonomous mechanisms [2–5]. In *Arabidopsis,* the vernalization genes are induced by low temperature over the cold period, and this leads to suppressing *FLOWERING LOCUS C (FLC)* that represses the floral transition [6, 7]. The photoperiod mechanism consists of the photoreceptors and the circadian clock [8] that involves two primary genes *CONSTANS (CO), and FLOWERING LOCUS T (FT)* [9]. During the light period, *CO* is overexpressed, resulting in the activation of *FT* which acts as mobile florigen that is expressed in leaves, moves through the phloem to reach the shoot apical meristem, and activates floral identify genes *APETALA1 (AP1)* and *LEAVES FLY (LFY)* [10, 11]. The endogenous growth regulator GA upregulates the transcription of *SUPPRESSOR OF OVEREXPRESSION OF CO1 (SOC1)* known as an activator of *LFY* [12]. In monocotyledonous plants, flowering time regulation has been intensively investigated in most economically important crops such as maize, rice, barley, and wheat*,* for which, vernalization*,* photoperiod*,* and earliness per se pathways were identified [13, 14]. For winter wheat, vernalization induced *VRN1* (ortholog of *AP1*) that expresses in leaves and acts as a repressor of *VRN2* (ortholog of *FLC*) which promotes the transcription of *VRN3* (ortholog of *FT3*) when days get longer in spring [15, 16]. The photoperiod pathway in wheat is regulated by homoeo-allelic gene series *PPD*, which encodes a pseudo-response regulator (*PRR*) family protein gene orthologous to the *Arabidopsis PRR7* gene. Wheat *Heading date 1 (TaHD1)* gene is the homolog of *CO* in wheat and exhibits diurnal rhythm (peak during the day, low at night) under long days [17]. In wheat, *PHYTOCHROME C (PHYC)* is the elementary light receptor that transmits light input to the photoperiod pathway, by promoting the transcription of *PPD1* and accelerates flowering via *VRN3* in long days [18]**.** Earliness per se, which corresponds to the autonomous flowering pathway in *Arabidopsis* involved genes such as *Eps-3A*^*m*^ gene of *Triticum monococcum* which is an orthologue of the *Arabidopsis LUX/PCL* gene [19] and *Eps-1A*^*m*^ related to wheat *ELF3* gene [20]. It was reported that many *Eps* genes are active in a temperature-dependent manner, correspond to components of the circadian clock, and mediate light signaling [21, 22]. Phytohormones such as ABA, CK, Ethylene, and Brassinosteroids contribute to the flowering process in *Arabidopsis* [23–25]. Thus, exogenous and endogenous floral integrators crosstalk with each other and channelize the signals via several regulatory elements to control the floral switch.

To identify genes underlying complex traits, quantification of gene expression levels using RNA sequencing (RNA-seq) analysis is a powerful technique to achieve this goal [26]. In plants, RNA-seq was exploited to investigate biotic and abiotic stress resistance [27], tillering [28], flower development [29], and fruit formation [30]. The transition to the reproductive phase was subject to large-scale transcriptome analyses in many important cereal crops such as maize [31], rice [32], barley [33], and wheat [34]. RNA-seq has also proven to be a time and cost-effective method for detecting single nucleotide polymorphisms (SNPs) in transcribed genes and consequently analyzing the allele mining that harbors a target locus [35]. The identification of such genomic loci and their related SNPs resulting from natural variation and account for significant phenotypic alteration of a given trait is the ultimate target of genome wide association studies (GWAS) [36]. Despite the high reliability of GWAS, it does not lead necessarily and directly to the gene(s) responsible for phenotypic variation because of insufficient marker density and/or decay of linkage disequilibrium in some cases. Combining QTL mapping with analysis of RNA-seq data to improve the interpretation of GWAS results has previously proven to be efficient in plant-based studies [37–39], Especially in studies dealing with flowering time in Brassica species [40, 41] and maize [42].

Pre-anthesis (heading) development in cereals is divided into three distinctive phases based on the morphological changes of the shoot apical meristem: the vegetative phase, the early reproductive phase, and the late reproductive phase [43]. Waddington et al. [44] developed a quantitative and developmental scale that describes the morphogenesis and progression of the shoot apex and carpels.

In this study, we joined QTL mapping provided by previous GWAS to transcriptome sequencing analysis for identifying candidate genes underpinning the detected QTL that underlay flowering time regulation in winter wheat [45]. For that, the contrasting genotypes identified in [45] are selected to perform comparative transcriptome analysis. The particular goals of the current study were to (1) assess the correlation between the observed flowering time trait in the field with microscopical phenotyping of trait-specific organ and stage, (2) to identify and map the genes differentially expressed in the early and late flowering cultivars in trait-specific organ and stage, (3) to explore the pathways and responses revealed by RNA-seq in QTL intervals and finally (4) to compare transcription levels of some selected genes mapped in significant QTL with relative gene expression via RT-PCR and identify polymorphisms in coding sequences and promoter regions of those genes.

## Material and methods

### Plant material

For the transcriptome study, two bred winter cultivars developed in Germany showing contrasting and stable flowering behavior in different environments (Additional file 1) were selected. The mean value of the heading date (HD) of both cultivars is based on the phenotyping data collected from six locations across Germany over 3 years [45].  “Kontrast” is the earliest flowering one in the adapted cultivars, which is released in 1990, and flowers 10 days earlier than the latest flowering cultivar “Basalt”, developed in 1980 [46]. The Australian cultivar Triple dirk “S”, which flowers 5 days earlier than “Kontrast” in the field, is cultivated since 1968 and was used as control.

### Plant growth conditions

The seeds of the selected extreme genotypes were sown in 96-well growing plates and kept in the greenhouse over 2 weeks for germination at 18 °C. Subsequently, the plants were transferred to a climate chamber to vernalize for 8 weeks in short-day conditions (8 h light at 22 °C and 16 h dark at 18 °C). Then, the plants were shifted to long-day conditions (14 h, 22 °C light; 10 h, 18 °C dark) until flowering.

### Microscopical phenotyping of shoot apical meristem

The phenotyping of the shoot apical meristem SAM was performed by dissecting the plants every 2 days after vernalization. After removing the leaves covering the floral organ, the apex was cut very quickly using a microsurgical disposable blade under a binocular microscope to avoid dehydration of the apex. The development of SAM was observed using the digital microscope KEYENCE model VHX-900F (KEYENCE Corporation, Osaka, Japan). The morphogenetic advancement of SAM was determined according to the developmental scale as described by Waddington et al., [44].

### Statistical analysis

The phenotypic data were compared between all cultivars by running a paired student’s t-test. Significance was compared with *p-*value < 0.01. The regression slopes were calculated in excel.

### Tissues collection for RNA analysis

The SAM and leaves materials were collected at three Waddington stages (W): W1.25-W1.75 (transition apex phase TAP), W2.0-W2.5 (double ridge stage DRS), and W3.0-W5.0 (late reproductive phase LRP), which correspond to time points 5, 13, and 25 days after the end of vernalization (DAV**)**. Depending on the development stages of each cultivar at the time of collection, the pooling of 20 to 60 shoot apices was needed to reach the minimum weight of tissue required for RNA extraction. We strictly selected shoot apices that showed a uniform morphological development per time point. The distal part of leaves samples was harvested at the same time points as mentioned above and from the same plants from which SAM was collected. For each cultivar, three biological replicates were collected. The samples were frozen immediately in liquid nitrogen and stored at − 80 °C.

### RNA-seq analysis and data processing

Total RNA extraction from the collected tissues, initial quality control, and sequencing analysis were performed commercially at Novogene Co. Ltd. (HK, China). Considering two cultivars * two tissues * three-time points * three biological replications, 36 libraries were constructed and sequencing based on the sequencing platform NovaSeq 6000 (Illumina) using the sequencing strategy paired-end 150 (=PE150) yielded on average 52.76 million 100 bp paired-end reads per sample. We used the RAW-ABS workflow for automated quality control and preprocessing of the RNAseq reads (https://github.com/tgstoecker/RAW-ABS/tree/v1.0; DOI: 10.5281/zenodo.3865747). Quality assessment of reading libraries was performed using FastQC v0.11.8 and Trimmomatic version 0.3 [47] to remove low-quality reads and remaining adapter sequences from each dataset. Specifically, a sliding window approach was used, in which a read was clipped if the average quality in a window of four bp fell below a Phred quality score of 20. BBDuk of the BBTools suite (https://jgi.doe.gov/data-and-tools/bbtools/) was employed to remove rRNA reads from the datasets using a kmer length of 27 as filtering threshold for decontamination. The splice-aware STAR aligner v2.7.3a [48] was used to align the remaining reads against a genome index of the bread wheat reference sequence and annotation - IWGSC “RefSeq v1.0” & “RefSeq Annotation v1.1” [49]. Multi-mapping reads that mapped to more than one position were excluded from subsequent steps by considering only reads, which mapped in a single location (outFilterMultimapNmax 1). On average, 50.8 million reads per sample aligned to unique positions in the gene set of the RefSeq v1.0 wheat reference genome with 120,744 predicted coding and non-coding gene models (EnsemblPlants release 46 [50]). The aligned paired-end reads were ordered according to their position and transformed to bam files with the software samtools version 1.9 [51]. We employed feature Counts v1.6.4 [52] to obtain aggregate counts of aligned reads at exon-level and to construct a gene-level matrix of these counts comprising all samples. The transcripts have been mapped in the previous four identified QTL for heading [45]. The list has been extended to 23 QTL that are statistically significant to explore as much as possible the pathways and responses revealed by RNA-seq (Additional file 2).

### Differential gene expression analysis

Differentially expressed genes DEGs were identified with the package “edgeR” version 3.26.4 [53] using the R language [54]. Differential expression analysis was based on comparing DEGs between the genotypes at the three-time points. Only genes passing a false discovery rate FDR < 0.05 and a |log2FC| > 1 were considered differentially expressed.

### Gene ontology term and pathway enrichment analyses

We performed de-novo functional annotation of the RefSeq v1.1 gene models with human-readable descriptions, including GO terms using AHRD (manuscript under review; https://github.com/groupschoof/AHRD).

GO functional enrichment analysis was conducted using the R package topGO [55] using the weight01 algorithm and a *P*-value threshold of ≤0.05.

### RNA extraction, cDNA synthesis, and gene expression analysis

Total RNA extraction from SAM and leaves was performed using RNAeasy Plant Mini Kit (Qiagen, Hilden, Germany, following the manufacturer’s instructions by using 100 mg tissue. The obtained RNA was subsequently treated with DNase to remove possible DNA contaminations using my-Budget DNase I (Krefeld, Germany, Bio-Budget Technologies). The quality of RNA was visualized by gel electrophoresis on 1% of agarose gel and quantified with a Spectrophotometer (ND-1000 Spectrophotometer, NanoDrop Technologies, USA). cDNA was synthesized from 1 μg total RNA using RevertAid First Strand cDNA Synthesis Kit (Thermo Scientific, Waltham, MA, USA) according to the manufacturer’s instructions. The possible contamination of cDNA with DNA was checked via PCR by *TaActin* gene (TraesCS1B02G283900) using designed primers flanking an intron (5′-CCATCATGAAGTGTGACGTGG-3′, 5′-TCCAAGGATGAGTACGACGAG-3′, Ta = 58 °C), The quantification of expression levels of the target genes was performed by RT-qPCR using DyNAmo ColorFlash SYBR Green qPCR Kit (Thermo Fisher Scientific Inc., Massachusetts, USA) and Applied Biosystems 7500 Real-Time PCR System (Life Technologies, Carlsbad, CA, USA) following the manufacturer’s instructions. The *TaEf-1.2* gene [56] was used as an internal control*.* The average Ct values of three technical replicates per reaction were calculated and used as input to estimate the expression of the target genes relative to *TaEf-1.2* using the 2 − ^ΔΔCT^ method [57]. The primers used in RT-qPCR for each selected gene are listed in Additional file 3.

### Analysis of promoter region and coding sequence of candidate genes

The amplification of the promoter region and coding sequence of targeted candidate genes was performed via PCR. For this, DNA from cultivars “Kontrast”, “Basalt”, and control was extracted following the protocol of DNeasy Plant Mini Kit (Qiagen, Hilden, Germany). The PCR amplification reactions were performed in a 25 μL reaction volume containing 100 ng of genomic DNA, 1 ^x^ One Taq standard buffer 10 μM of forward and reverse primers each, 0.2 mM of dNTP, and 0.5 unit of Taq DNA polymerase (NEB, Frankfurt, Germany). The PCRs were conducted in the thermocycler Flex cycler (Analytik GmbH, Jena, Germany). PCR profiles were visualized by electrophoresis on a 1% agarose gel stained with peqGreen (0.04 μl/mL; VWR, Darmstadt, Germany). The obtained PCR products were purified using the Purelink Quick PCR kit (Invitrogen, Waltham, MA, USA) and after undergoing sequencing from both ends. The primers used for PCR and Sänger-approach-based sequencing are listed in Table S1. The sequencing was carried out by Eurofins Genomics GmbH (Ebersberg, Germany). The obtained sequence information was then in silico analyzed to identify specific motifs and transcription binding sites (TBS) within the promoter region using PlantTFDB v5.0 [58]. The alignment of sequenced coding regions was performed using the MegAlign Pro tool of DNASTAR software (DNASTAR. Madison, WI). Identification of putative start and stop codons and exons-introns regions was carried out using the Ensembl database (http://plants.ensembl.org).

## Results

### Morpho-histological phenotyping of shoot apex development at the transition phase

**Fig. 1:**
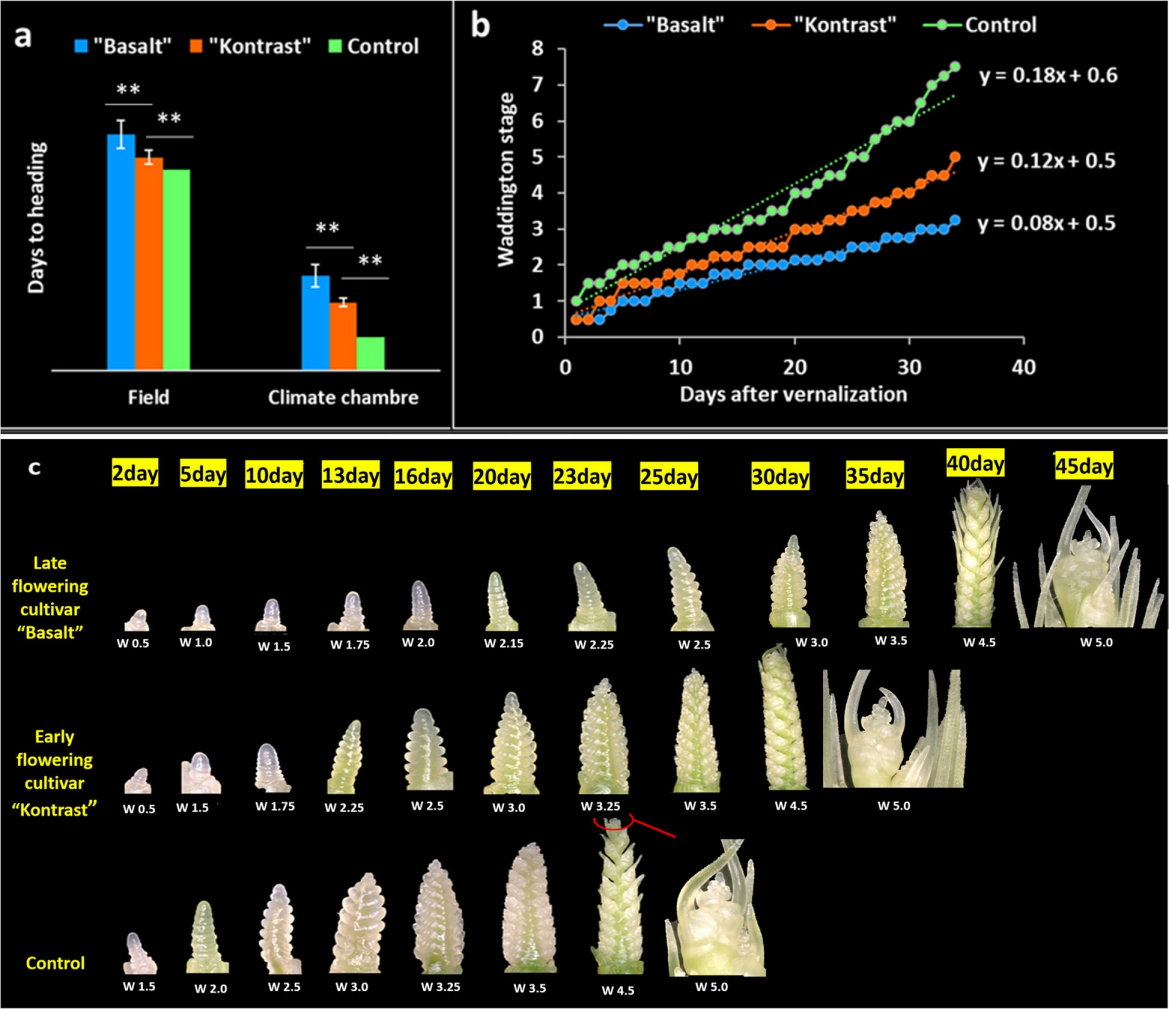
Comparative microscopical development of shoot apical meristem of two adapted cultivars “Basalt” and “Kontrast” showing late and early heading time. **a** Days to heading scored in the field and the climate chamber for the control, “Kontrast” and “Basalt”. ** Significance at < 0.01 of the probability level. **b** Regression analysis of shoot apex development after vernalization of control, “Kontrast” and “Basalt” according to Waddington scale. **c** Microscopical description of main shoot apex development of control, “Kontrast” and “Basalt” from day 2 to day 25 after vernalization

To investigate the heading shift observed in the field between cultivar “Kontrast” and “Basalt”, a comparative analysis of the SAM morpho-histological development was performed. The climate chamber conditions accelerated significantly (*P* < 0.01) the days to heading by 93.5, 81.2, and 65.6 days for cultivars “Basalt”,  “Kontrast” and control, respectively (Fig. 1a). HD range moved from 10.4 in the field to 12.3 days between the early and late adapted cultivars, while the control headed 8 days earlier than “Kontrast”. In the field and under climate chamber conditions, the same heading behavior and ranking were observed. The quantitative development of shoot apex revealed distinguishable SAM progresses observed in the three cultivars without overlapping at any Waddington stage. Paired student’s t-test showed differences between Waddington scores of SAM development in the three cultivars during the observation phase that extended to 35 DAV (Additional file 4). “Basalt” showed the slowest SAM growth compared to “Kontrast” and control. The slopes of regression lines were 0.08, 0.12, and 0.18 for “Basalt”, “Kontrast” and control, respectively (Fig. 1b). The microscopic phenotyping of SAM showed that the DRS was reached by “Basalt”, “Kontrast” and the control approximately at 25, 13, and 5 DAV, respectively. The shoot apex persisted in the vegetative phase (W0.5-W1.0) in “Basalt” until day 10. Then, the slow transition to the DRS lasted 15 days. The control moved very early to TAP on day 2, which needed only 5 days to reach DRS, while “Kontrast” took 13 days to reach the same stage (Fig. 1c). The days 5, 13, and 25 after vernalization were considered for further analysis.

### Description of transcription variants in leaves and shoot apex of early and late flowering cultivars

**Fig. 2:**
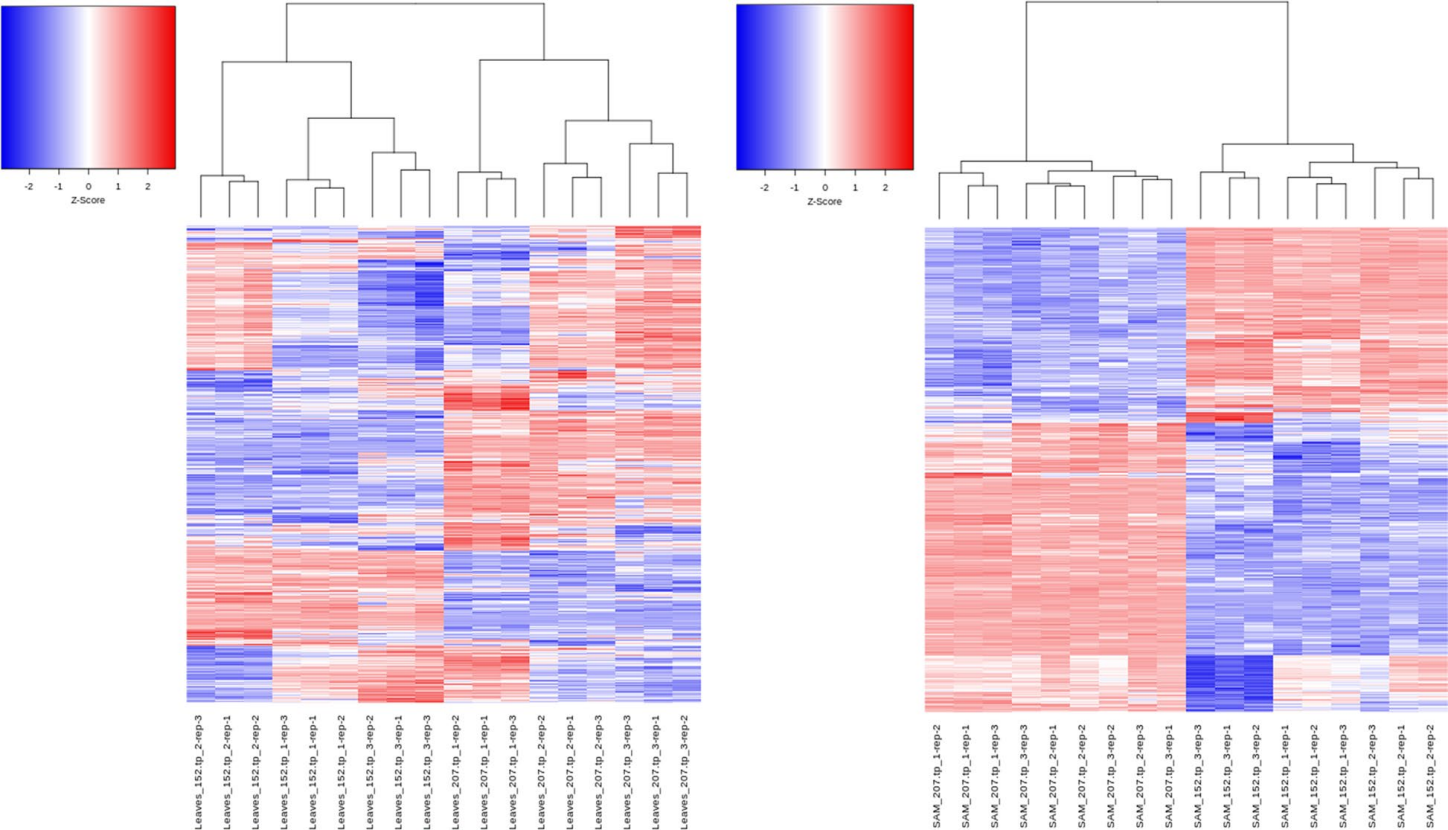
Hierarchical clustering of mapped and annotated DEGs in “Kontrast” and “Basalt” in SAM (right) and leaves (left). Z-score represents the standard deviation from the mean value of all samples. Samples are clustered, based on the Euclidean distance between the expression values of the samples

To identify candidate genes responsible for the floral switch, we conducted whole-transcriptome expression profiling of SAM and leaves of the two adapted early and late flowering cultivars in three selected time points. Counting only mapped and annotated genes, RNA-sequence analysis of 36 libraries yielded 10,532 DEGs in SAM **(**Additional file 5**)**, 31, 18.4, and 50.6% were found in time points 5, 13, and 25 DAV, respectively. In leaves, 16,007 DEGs remained **(**Additional file 6**)**, 33.3, 21.1, and 45.6% were distributed in time points 5, 13, and 25 DAV, respectively**.** The hierarchical clustering revealed more closeness between the three biological replicates per cultivar and time point in SAM than in leaves. Transcriptional changes between time points occurred more frequently in leaves and the DEGs that showed higher expression levels than the average were more observed both more frequently in leaves as well. The number of positive high expression levels relative to average is greater in “Kontrast” than “Basalt” when considering the apex tissue (Fig. 2).

### Mapping the expressed flowering time regulators in the QTL intervals

**Fig. 3:**
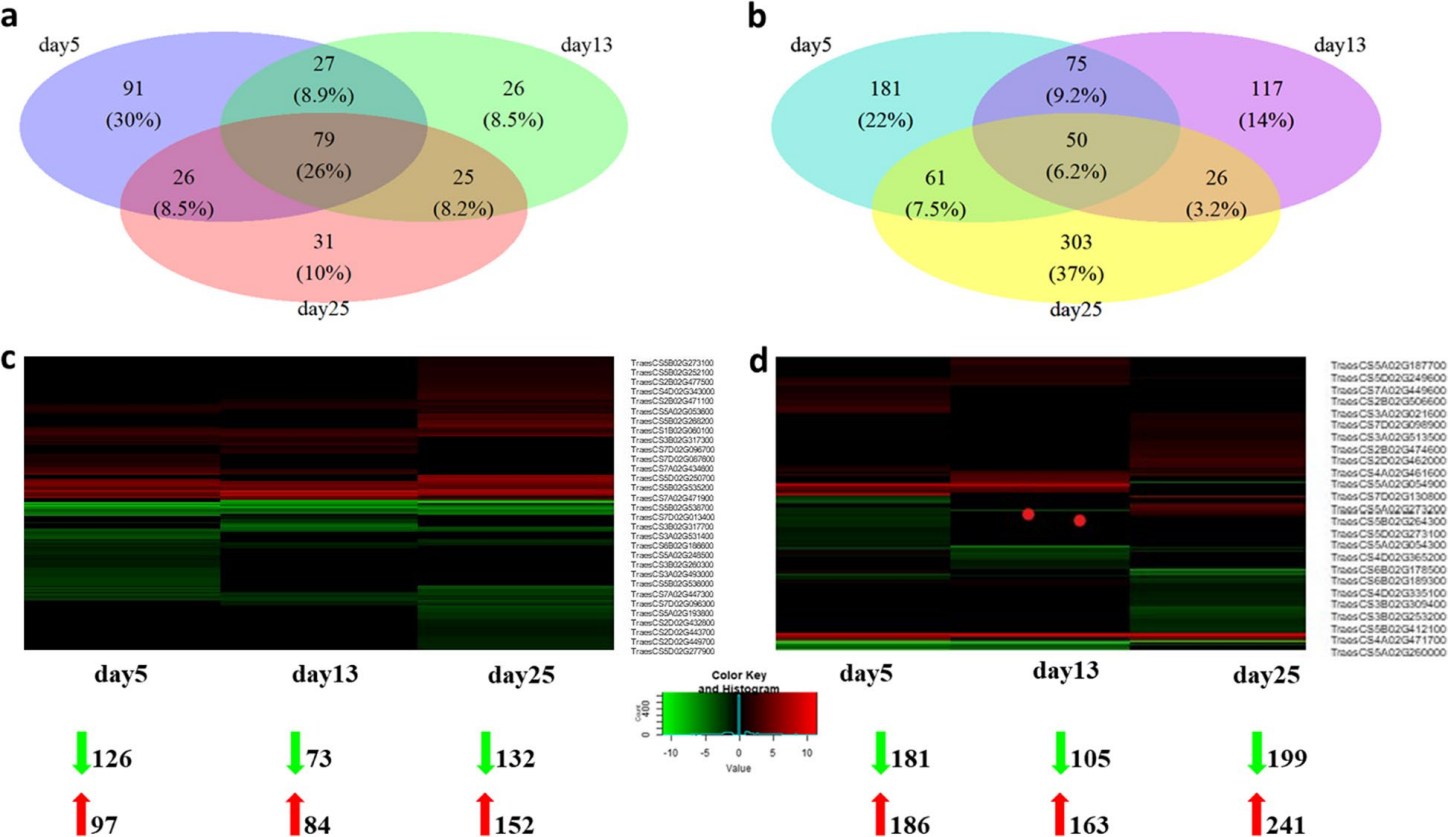
Differential gene expression analysis in main shoot apex and leaves mapped in 23 QTL intervals associated with flowering time trait. **a** and **b** Venn diagrams showing the number and percentages of mapped DEGs in the early flowering “Kontrast” relatively to the late one “Basalt” in 5, 13, and 25 DAV in SAM and leaves, respectively. **c** and **d** Heatmap for visualization of the regulation pattern of mapped DEGs based on fold change estimation between “Kontrast” relatively to “Basalt” in 5, 13, and 25 DAV in SAM and leaves, respectively. The mean value of Log_2_ FC includes three biological replicates. Genes not passing FDR < 0.05 Fold and change > (±) 2 were set to value =0 (black). The number of upregulated DEGs (red) and downregulated ones (green) are shown at the bottom

The goal was to determine the genes involved in the transition from the vegetative to reproductive phase. For that, we applied a strategy to combine genetic analysis with comparative transcriptomics. The previously four uncovered loci involved in the regulation of flowering time detected in adapted German wheat cultivars [45] plus 17 other significant QTL were used for downstream selection of DEGs comparing “Kontrast” to “Basalt”. In total, 670 and 1075 genes were differentially expressed between the cultivars in SAM **(**Additional file 7**)** and leaves **(**Additional file 8**)**, respectively, and could be mapped to the 23 significant QTL intervals. The TAP involved 91 DEGs in SAM and 181 in leaves. In all, 26 DEGs were specific to 13 to DRS in the early flowering “Kontrast” at SAM (31) during the change to the LRP **(**Fig. 3a, b**).** By contrast, 26% of total DEGs in SAM were co-regulated during all time points, while only 6.2% of genes were continuously regulated in leaves samples. For both organs, the DRS yielded less number of DEGs in comparison to vegetative and reproductive time points. The visualization of DEGs regulation revealed the same three patterns of expression in SAM and leaves: stable up/downregulation in all-time points, up/downregulation in one and two-time points **(**Fig. 3c, d**).** The |log_2_FC| which indicates the log-ratio of a gene’s expression values ranged from − 11.3 for downregulated DEGs to + 8.9 for upregulated ones.

### GO enrichment analysis of DEGs in the apex and leaves

Gene Ontology (GO) terms were assigned to DEGs to functionally characterize developmental and SAM and leaf responsive processes and functions. Overrepresented functional categories in each time point in the QTL intervals were identified by gene ontology (GO) enrichment analysis in the early flowering “Kontrast” relatively to “Basalt” (*p* < 0.05)**.** Fill lists of enriched GO terms in the three analysed time points in SAM and leaves is provided in Additional files 9 and 10, respectively, where their visualisations are shown in Additional file 13. Comparison of the ontologies in SAM tissues showed that the highest number of GO-terms are assigned at LRS stage with particularly higher proportions of ontology in the category of biological processes (53%) and molecular functions (40%). Among functional terms, that showed upregulation are the histone H3-K9 demethylation (GO: 0033169) at both TAP and DRS. Other terms include regulation of abscisic acid-activated signaling pathway (GO: 0009787), euchromatin (GO: 0000791), protein kinase activity (GO: 0004672), whereas downregulated DEGs assigned in terms like regulation of salicylic acid biosynthetic process (0080142), positive regulation of auxin metabolic process (GO: 0048621), positive regulation of long-day photoperiodism, lowering (GO: 0048578), for plant hormones like negative regulation of cytokinin-activated signaling pathway (GO:0080037), negative regulation of brassinosteroid mediated signaling pathway (GO: 1900458). The terms assigned in leaves show different pattern than in SAM (Additional file 13) except a few terms that are in common. One of them is for example the histone H3-K9 demethylation (GO: 0033169). These findings might indicate that the transcription of genes in vegetative and reproductive tissues is differentially regulated.

### Organ-specific genes at the transition phase detected in QTL intervals

Among the 91 DEGs specific to the TAP in SAM **(**Fig. 3**),** three GO terms are related to flowering time: histone *H3-K36* demethylation, CK transport, and regulation of circadian rhythm **(**Additional files 7 and 9**)** Histone *H3-K36* methylation is represented by three homoeologous genes on chr 5: *TraesCS5A02G265500*, *TraesCS5B02G265200,* and *TraesCS5D02G273400* mapped in QTL TaHd112, TaHd124, and TaHd137, respectively. These genes, coding for the *CUPIN-LIKE* domain are also associated with the regulation of circadian rhythm. Far-red light phototransduction involves two genes *TraesCS3B02G318600* and *TraesCS5B02G422000* mapped in QTLTaHd054 and TaHd129, annotated as *SPA1-RELATED 3* and transcription factor *PIF5,* respectively. The response to temperature could be detected in leaves tissue as well via the gene *TraesCS5A02G260600* from QTL TaHd112, which encodes a *HEAT SHOCK* protein **(**Additional files 8 and 10).

The 26 DEGs, identified specifically in the DRS in the apex, are clustered in four significant (*p* < 0.05) GO terms: G-protein coupled receptor signaling pathway, monovalent inorganic cation homeostasis, regulation of the cellular biosynthetic process, and plant-type cell wall modification. Blasting all genes of those pathways led to uncovering the gene *TraesCS3B02G318300* found in QTL TaHd054, which controls the regulation of floral organ identity via MADS-box transcription factor 32. Simultaneously, ethylene regulation is triggered in the leaves because of the expression of *ETHYLENE INSENSITIVE 3* related to gene *TraesCS5B02G265400* (QTLTaHd124) and its homoeologous *TraesCS5A02G265700* (QTLTaHd112). Under the regulation of stomatal movement GO:0010119, the gene *TraesCS7D02G111600* annotated as *FLOWERING LOCUS T* is mapped in the last QTL TaHd177 on chr 7D.

*GLYCOSYLTRANSFERASE* protein encoded by two genes *TraesCS2D02G462500* and *TraesCS3B02G313500*, mapped in loci TaHd038 and TaHd054, respectively, expressed exclusively in the apex at LRP. At this stage, three homoeologous genes *TraesCS5A02G264800*, *TraesCS5B02G264300* and *TraesCS5D02G272800*, localized in loci TaHd112, TaHd124 and TaHd137, respectively, encode the transcription factor *bHLH130* classified under photoperiodism and flowering (GO:0048573). The response to red and far light (GO: 0010114) was detected in the form of transcription factor *PIF3* encoded by *TraesCS2D02G461700* from QTL TaHd038. In leaves tissue, many genes expressed at LRP and related to the circadian clock (GO: 0042752) could be mapped in the identified QTL. For instance, *TraesCS7A02G431600* (TaHd166) and *TraesCS3A02G526600* (TaHd049) encodes *ADAGIO-LIKE* protein and *LUX/PCL1*, respectively. While, *TraesCS4A02G474100* (TaHd073) and *TraesCS7A02G470700* (TaHd166) encode the same Protein *REVEILLE 6 (RVE6).* The expression of the transcription factor *ASYMMETRIC LEAVES (AS1)* was reported to respond to Gibberellin acid encoded by the gene *TraesCS5A02G079100* mapped in the QTL TaHd102.

Among the genes mapped to QTLs that are consistently regulated in the three phases of the floral switch, 547 expressed and 150 GO annotated genes were found shared between SAM and leaves in at least one stage. In this category, *FRIGIDA-like* protein could be identified as a transcription product of the gene *TraesCS5B02G543400* localized in locus TaHd132. *FRIGIDA-like* protein is detected as well at TAP and DRS in the leaves. Many transcription factors were permanently controlled as a response to light such as light-inducible protein *CPRF2* encoded by the genes *TraesCS5A02G057500* (TaHd098) and *TraesCS6B02G182500* (TaHd152) found both in SAM and leaves. In leaves, mRNA cleavage and polyadenylation specificity factor are related to the gene *TraesCS5B02G536400* (TaHd132) and expressed in the three phases. The response to CK (*TraesCS4A02G228800*, TaHd071), ABA (*TraesCS5A02G069500*, TaHd099), Auxin (*TraesCS5A02G058700*, TaHd098) and other numerous continuously expressed regulatory transcripts related to glucose, metal (nitrate, iron, zink, and cadmium), phosphorylation, and fatty acid could be mapped in QTL intervals in SAM and leaves **(**Additional files 9 and 10).

### RT-qPCR expression analysis of selected flowering time candidate gene *AS1*

**Fig. 4:**
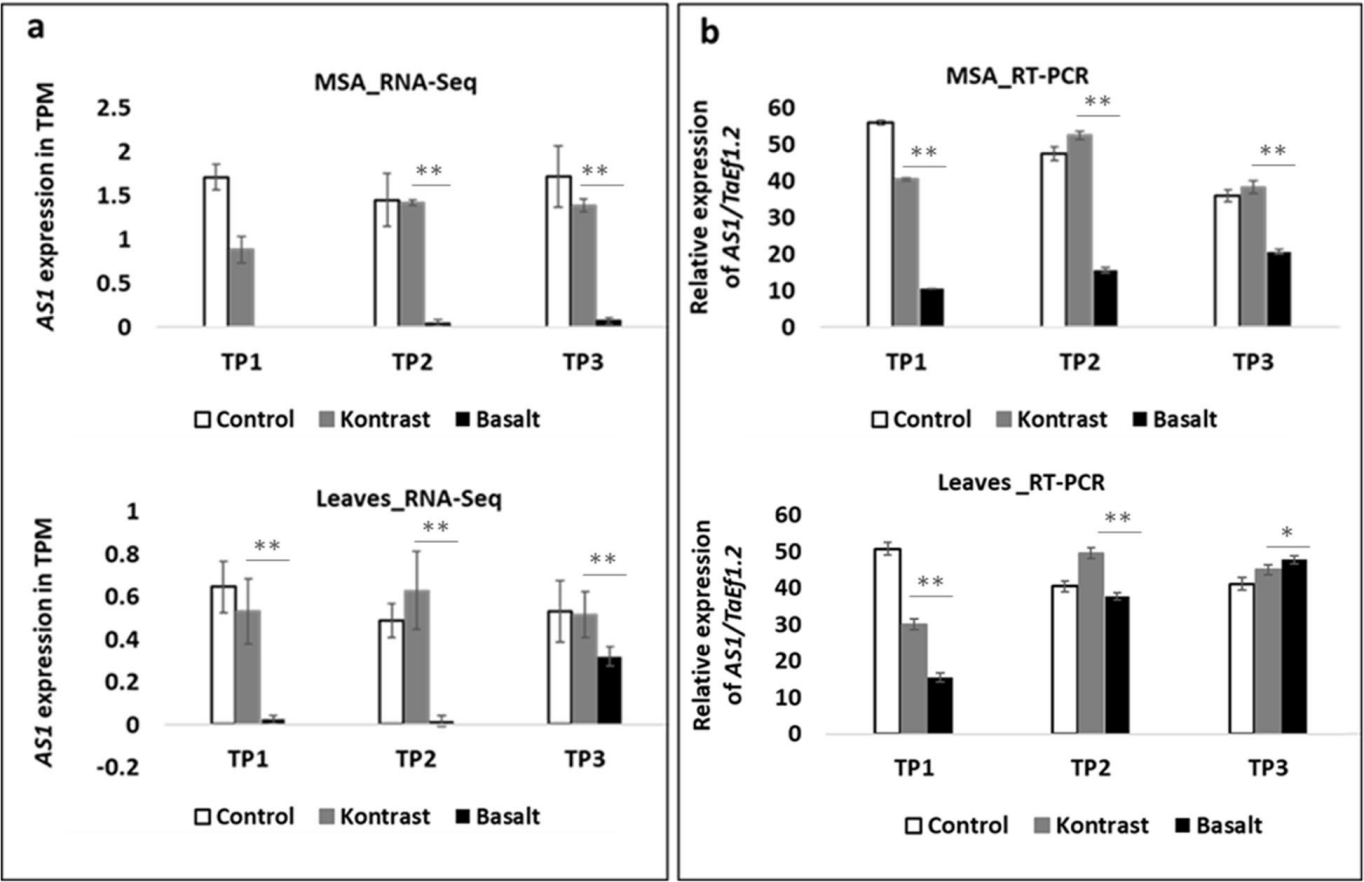
Expression of *AS1* using RT-quantitative PCR in SAM and leaves for the three-time points: TP1 (TAP), TP2 (DRS), and TP3 (LRP) in “Kontrast”, “Basalt” and the control. **a** Gene expression of *AS1* in TPM (Transcripts per million in SAM (up) and leaves (down) using RNA-seq output. **b** Expression of *AS1* relatively to the internal control *Ta.Ef1.2* in %. *, ** Significance at the 0.1 and 0.01 probability levels, respectively

To check the reliability of the RNA-Seq data**, s**ix DEGs (three from each cultivar/time point) were randomly chosen for verification via qRT-PCR. The results showed that the relative gene expression levels of the selected DEGs were consistent with expression profiling resulting from the RNA-seq analysis **(**Additional file 11). One locus TaHd102 (98.3–125.1 Mbp) mapped on chr 5A, showing a high association to flowering time trait (*P* < 0.0001) [45], was used for further analysis of DEGs as inferred from the RNA-sequencing data. TaHd102 bears the gene *TraesCS5A02G079100* (98.4 Mbp), encoding the transcription factor *AS1*, which was selected for gene expression analysis using RT-quantitative PCR in SAM and leaves for the three-time points in the early “Kontrast”, late “Basalt” and the control (Fig. 4a and b). In SAM, The analysis revealed that *AS1* reached its maximal expression in the control in TP1 and TP2, in “Kontrast” in TP2 and TP3, and in “Basalt” in TP3. The same expression pattern was observed in leaves, where the expression level of *AS1* in the late “Basalt” at TP3 is closer to the expression level in “Kontrast” and the control when they reached the DRS than in SAM. The RT-qPCR results are almost in line with RNA-seq expression profiles with more similarity in leaves than in SAM. *AS1* expression in SAM could not be detected via RNA-seq in “Basalt” at TP1 and showed very low levels at the other time points for the same cultivar. Comparing only “Kontrast” and “Basalt”, the fold change of differential expression of *AS1* in “Kontrast” relatively to “Basalt” is much higher in RNA-seq output than in RT-qPCR.

### Promoter region analysis of transcription factor *AS1*

**Fig. 5:**
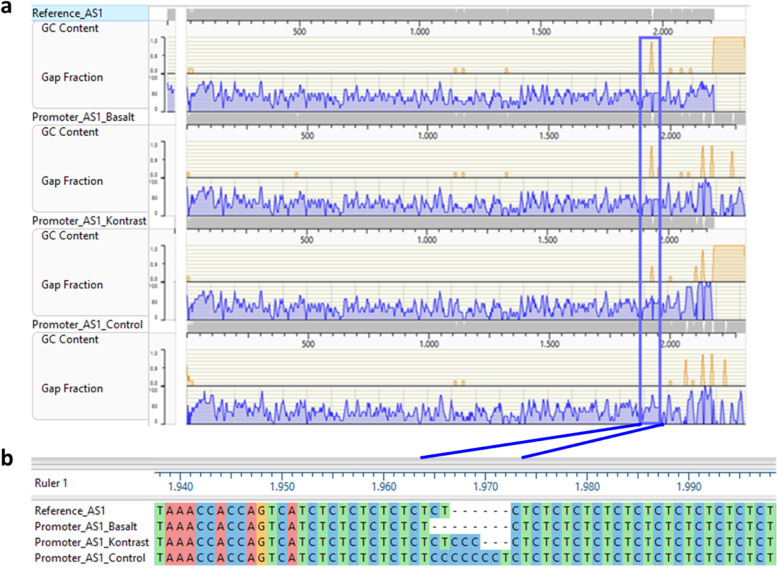
Alignment of *AS1* promoter region (2 kb) sequence. **a** In cultivars “Basalt”,  “Kontrast” and control compared with the reference sequence from EnsemblPlant database. GC content and Gap fraction are indicated in blue and orange lines, respectively. **b** Comparison of the motif sequence of the TFBS CTCTCTCCCCCCCTCTCTCTC in the three cultivars and the reference sequence at positions 1972–1966 bp

The promoter region of the *AS1* gene is highly conserved and 96% of the sequenced 2 kb upstream of the start codon is similar in all cultivars **(**Additional file 12**)** that share 94.8% of the conserved TF binding site of their 2 kb promoter regions. The alignment output uncovered a deletion of eight single nucleotides in “Basalt” between positions 225 and 231 upstream of the translation initiation site compared to control. In the same region, “Kontrast” revealed a deletion of only three single nucleotides in its promoter region from the same region detected in the control (Fig. 5). This polymorphism is followed by one SNP (C/T) at position 232 where nucleotide T in the two adapted cultivars “Kontrast” and “Basalt” is substituted by C in the exotic control. The sequence TCCCCCCCTCTCTCTCTCTCT (http://planttfdb.gao-lab.org/tf.php?sp=Tae&did) is the core motif of the TF *Traes_1AL_6B108514B* from *MADS BOX* TF family.

## Discussion

### Assessment of flowering behavior by microscopical phenotyping of the main shoot apex

In this study, the earliest and latest flowering cultivars “Kontrast” and “Basalt” were subject to microscopical visualization of SAM development. This comparative analysis revealed the acceleration of the apex development of “Kontrast” compared to “Basalt” in the three phases of the transition from vegetative to reproductive stage, and consequently, asserts the early flowering behavior of “Kontrast” in the field. Furthermore, from the vegetative apex stage, no overlapping in SAM growth was observed between both cultivars during the floral monitoring, which means that the difference in progress rate from one stage to the next was stable between both cultivars; this is shown by comparing the regression slopes of SAM development after vernalization in “Kontrast” and “Basalt”. Before the DRS, the spikelets are induced at day 5 in the control, whereas the LRP arises in more than 15 days. Thereby, spikelets are initiated at a much faster rate than after the DRS. Many studies have reported that the dynamic of the floral initiation marked by the first spikelet primordium until the initiation of the last one is much accelerated compared to that of the terminal spikelet to anthesis (floret primordia) [59, 60]. The duration of the early reproductive phase determines the number of spikelet primordia initiated on the shoot apex [61, 62]. However, no significant difference in spikelet primordia counts (six to seven) was observed in the late flowering “Basalt” compared to the early one “Kontrast”, even when the reproductive stage lasted 25 days in “Basalt”. The number of fertile florets developed within the spikelets is defined in the LRP [62]. For this trait, the comparison between “Kontrast” and the control (flowers earlier than “Kontrast”) showed no relation in the duration of the LRP. This can be explained by the fact that the final number of fertile florets is depending more on the number of florets that survived the degeneration and death mechanisms after floret initiation than on the duration of floret formation [63, 64]. On the other hand, the switch to constant long days and ambient temperature conditions after vernalization reduced significantly the number of days to heading in all cultivars, including the control, compared to field conditions. This result leads to conclude that the response to environmental stimuli such as light, photoperiod, and ambient temperature has a quantitative nature, while the stable heading time range is due to established genotypic differences among cultivars.

### Histone methylation and light response regulate the transition apex phase

Several pathways were upregulated in SAM and leaves tissues to promote the switch from vegetative to TAP (5 DAV). For example, genes Histone *H3K36* methylation are detected in three homoeologous loci on chrs 5A, 5B, and 5D. *H3K36* was found to induce flowering by activating alternative splicing and plant plasticity to fluctuating ambient temperature in *Arabidopsis* [65] and rice [66]. Interestingly, the same homoeologous genes are involved in the regulation of circadian rhythm as well. Circadian clock and histone methylation are connected pathways. *H3-K36* was found to antagonize the binding of *Arabidopsis* clock repressor *TIMING OF CAB EXPRESSION(TOC1)* ensuring that repression occurred at the proper time during the day and night cycle (oscillation) via chromatin changes [67–69]. Light is a signaling cue that controls many aspects of plant growth including the induction of flowering [70]. Some expressed light signaling components were downregulated and mapped in two loci such as *SPA1 (SUPPRESSOR OF PHYA-105) -RELATED 3*/TaHd054 which reduces the persistence of *PHYA* signaling and function in concert with *PHOTOMORPHOGENIC1(COP1)* to suppress photomorphogenesis in the dark [71, 72]. The second gene*, PHYTOCHROME INTERACTING FACTORS 5* (*PIF5/*TaHd129) functions negatively in *PHY*-mediated pathways and reduces red light sensitivity [73]. S*PA* and *PIF-like* genes have not been functionally validated in temperate grasses thus far. The response to low light intensity stimulus was found to be downregulated at this stage as well. One gene annotated in wheat as “light-harvesting chlorophyll a/b-binding protein (*LHCB*)” is classified in very-low-fluence responses and involved in inhibition of hypocotyl elongation and promotion of cotyledon expansion [74] in *Arabidopsis*. Moreover, the response to low-fluence blue light represses a *Pirin-like* gene. *M*utant plants for this gene in *Arabidopsis* flower earlier than wild-type plants [75].

### *TaAGL14* activates the floral switch and SNP at *VRN3* represses it in the double ridge stage

**Fig. 6:**
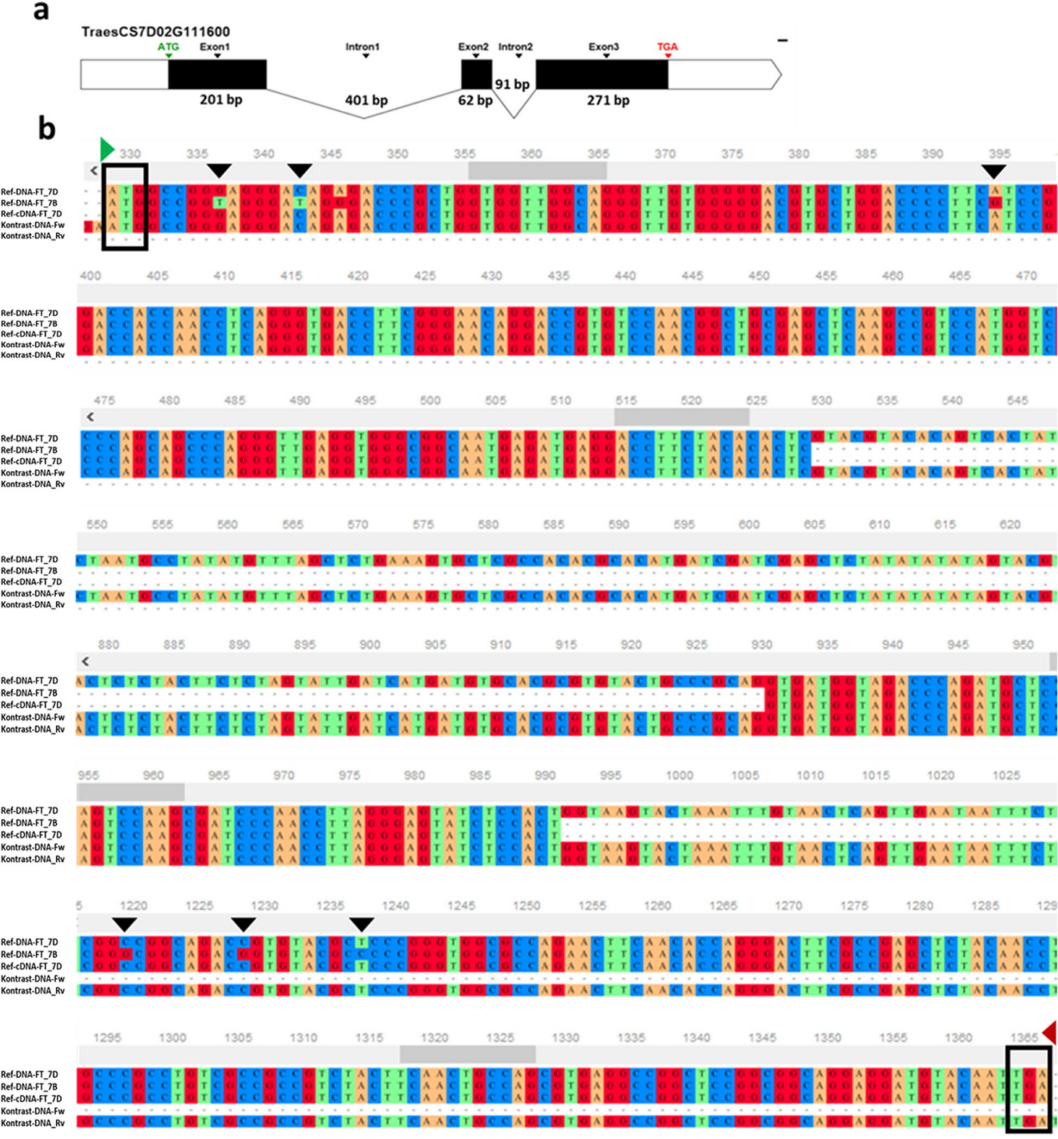
Coding sequence alignment of the gene *TraesCS7D02G111600* encoding *Flowering locus T.***a** Structure of the gene *TraesCS7D02G111600* mapped on chr 7D in early flowering cultivar “Kontrast” that contains three exons and two introns. **b** Alignment output of the gene *TraesCS7D02G111600* (forward and reverse sequences) with its homoeologous *(TaFT1)* mapped on chr 7B and the in Silico reference cDNA. Start and stop codon’s positions are indicated in green and red arrows, respectively. SNPs are highlighted with black arrows

**Fig. 7:**
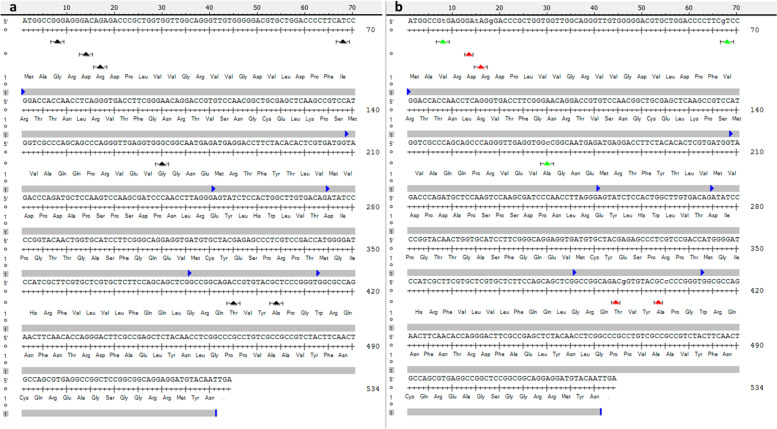
Translation of the *Flowering locus T* protein encoded by the gene *TraesCS7D02G111600.***a** Using the ORFs finder of DNAstar, Seqbuilder tool, the first ORF on top gave the longest and continued translation (176 amino acids). The alignment with the *VRN3* coding sequence lead to detect seven SNPs indicated with black arrows. **b** Effect of nucleotide substitution on amino acid change. Synonymous and non-synonymous SNPs are indicated by green and red arrows, respectively

The highlight result in the double ridge stage is the detection of the MADS-box transcription factor 32 in QTL TaHd054. MADSS32 wheat gene (*TraesCS3B02G318300*, SAM) is the ortholog of *OsMADS32* that regulates floral patterning in rice and takes charge of floral meristem identity and initiation through interactions with multiple floral homeotic genes to sustain floral organ development [76]. BLAST results showed that the predicted protein of *TraesCS3B02G318300* is identical by 99% with *TaAGL14*, 98% with *TaAGL15* in wheat*,* and 87% with *OsMADS32* in rice*.* Furthermore, *TaAGL14*, *TaAGL15,* and *OsMADS32* together, form a distinctive clade of *MIKC*-type gene family found only in grasses with no representatives from *Arabidopsis* [77, 78] reported the involvement of *TaAGL14* in stamen and pistils development in wheat. Here, we provide the first evidence about the function of the *TaAGL14* gene in an earlier reproductive stage in floral meristem activation in wheat, which may very likely be similar to *OsMADS32* function in rice. *TraesCS3B02G318300* was 4.5 fold more upregulated in “Kontrast” than in “Basalt”, which is in line with the activator role of *OsMADS32* in initiating the floral meristem and its role in the termination of floral meristem activity and repressing its reversion to vegetative meristem [76]. Ta*FT* (QTL TaHd177), from *Phosphatidylethanolamine*-binding protein (*PEBP*), was exclusively detected in the DRS in leaves tissue. Surprisingly, *TaFT1* transcription was strongly downregulated by log_2_FC = − 8.6 in the early “Kontrast” relatively to late heading “Basalt” cultivar. This fact contrasts with the well-documented function of *FT* as a floral promoter in *Arabidopsis*, rice, barley, and wheat. Actually, *FT* can be a floral repressor, too. It was reported that, because of gene duplication event(s), paralogs of *FT* with an antagonistic function were generated in sugar beet (*Beta vulgaris* L.) and tobacco (*Nicotiana tabacum* L*.*). In sugar beet, the first protein *BvFT1* acts as an inhibitor of the floral switch, whereas a second *FT-like* paralog protein *BvFT2* works as a promoter [79]. This is due to synonymous mutations in specific amino acids allowing the conversion of *BvFT1* to *BvFT2* and vice versa [79]. The tobacco genome harbors three *FT* floral inhibitors *NtFT1*, *NtFT2*, and *NtFT3*, and the fourth paralog *NtFT4* is a floral inducer [80]. The same phenomenon was discovered in *Arabidopsis* and tomato [81, 82]. This means, we may detect a copy of *FT* in wheat with the QTL effect showing an opposite function and acting as a floral repressor, which can explain the negative regulation of *FT* transcription in the early flowering “Kontrast” genotype. To examine this hypothesis we sequenced the coding sequence of the gene *TraesCS7D02G111600* (1026 bp) and performed an alignment of translated amino acids against the *TaFT1(VRN3)* protein on chr 7B (Fig. 6a and b). Among seven SNPs, three found in the first exon are synonymous, where a substitution of single nucleotide T/G leads to the change of the third amino acid valine to glycine. The second SNP G/A in the 23rd amino acid substitutes valine with isoleucine and the third SNP G/C in the position 56th coverts glycine into alanine (Fig. 7a and b). In wheat, the role of *TaFT1(VRN3)* on chr 7B is determined, while no validation of the homologs function on chrs 7A and 7D as floral inducers were reported so far. On chr 7D, two copies of *PEBP* are localized at 68.4 and 191Mbp (Ensembl plants database). As locus TaHd177 (63.5–73.8Mbp) includes the first copy (68.4Mbp), we tend more towards the supposition that the antagonistic player of *TaFT1* on chr 7B is very likely its homoeolog *TraesCS7D02G111600* on chr 7D mapped at 68.4Mbp. Further analysis is required to prove the responsibility of substituted amino acids in altering the role of the wheat *FT* from an inducer (*TaFT1* in 7B) into an inhibitor (*TaFT1* in 7D) in the flowering time pathway, as it is the case in many other plant species.

### Circadian clock is involved in hypocotyl and stem elongation in the reproductive phase

During the LRP, stem internodes elongate, and the floret primordia develop into flowers [44]. In this phase of spikelet development, some expressed flowering time key regulatory elements were mapped in QTL intervals. The transcription factor basic *HELIX–LOOP–HELIX* (*bHLH130*) was identified in three homoeologous loci on chrs 5A, 5B, and 5D. *bHLH130* annotated as *FBH4* (*AT2G42280*) binds to the E-box cis-elements in the *CO* promoter. The overexpression of *FBH4* strongly increases *CO* transcription and causes early flowering in *Arabidopsis* and rice [83]. This is in full agreement with our results showing differential upregulation of *bHLH130* in “Kontrast” by 5.4, 8.5, and 4 fold at loci TaHd112, TaHd124, and TaHd137, respectively. We conclude that the copy mapped in QTL TaHd124 on chr 5B has more effect than other homoeologous regions. *LUX/PCL1* belongs to clock players of the evening complex expressed in the night to regulate the nocturnal rhythmicity of the circadian clock [84]. Moreover, the *ELF4-ELF3-LUX* complex is regulated by the clock and light. It represses the expression of *PIF4* and *PIF5* required for hypocotyl growth in the early evening. *PIF4/5* regulation is turned over at dawn to permit maximal hypocotyl growth in *Arabidopsis* [85]. The expressed orthologue of *LUX/PCL1* in wheat was mapped in QTLTaHd049 and was by log_2_FC = 5.9 upregulated in “Kontrast”. We deduce that the regulation of circadian rhythm is more pronounced in the early flowering cultivar and this occurs in the late reproductive phase where the stem elongation initiates. We suggest that *LUX/PCL1* may be involved in the oscillator growth of the stem under circadian clock control as in *Arabidopsis*. QTL TaHd166 harbors the gene encoding *ZTL* orthologue in wheat and was found downregulated in “Kontrast”. This finding agrees with the reported results in *Arabidopsis* that over-expression of *ZTL* results in downregulation of *CO* and *FT* expression, leading to delayed flowering under long-day conditions [86, 87].

### Allelic variation in the promoter of *AS1* is associated with heading variability

QTL TaHd102 on chr 5A is strongly associated with heading date and explains 13.8% of the genetic variance observed in the German wheat germplasm [45]. *AS1* is the only annotated transcript in this locus known to be involved in the flowering time pathway in the *Arabidopsis* background. We conclude that the effect of QTL TaHd102 on heading variation is most likely due to the gene *TraesCS5A02G079100* encoding *AS1* protein*. AS1* is required for normal cell differentiation and leaves patterning by direct suppression of *KNOTTED-like HOMEOBOX (KNOX)* gene expression at leaves primordia in *Arabidopsis* [88, 89]. *KNOX* proteins repress the GA biosynthesis gene *AtGA20ox1,* thus *AS1* is possibly mediating the Gibberellin pathway [90]. In this study, RNA-seq and RT-qPCR confirmed the association of the expression of an *AS1* transcription factor with the early flowering time. This leads to conclude that the floral transition in wheat involves GA biosynthesis besides vernalization, photoperiod, and earliness per se. On the other hand*, AS1* forms a functional complex with *CO* to activate *FT* in photoperiodic *Arabidopsis* as reported by Song et al [91]*.* We found that transcription factor *bHLH130 (FBH4)* is strongly upregulated in the early flowering “Kontrast” and this *TF* binds directly to the E-box cis-elements in the *CO* promoter. We have no evidence that *AS1* is interacting with *CO,* as is the case in *Arabidopsis*; however, we provide first insight that *AS1* and *FBH4*, which activates *CO*, are inducing the floral switch and expressed both in leaves during the RP in wheat. In addition, the polymorphism in the promoter region of *AS1* in the studied cultivars concerns the core motif of the well-described *AGAMOUS-LIKE MADS-BOX* protein *AGL20* (*AT2G45660*) known as *SOC)*, which acts as an activator of flowering time in *Arabidopsis* [92] and rice as well [93]. *SOC1* expression is induced as a response to GA [12] by integrating the signals from vernalization [94] and photoperiod [95] in *Arabidopsis*. In light of that, we deduce that the deletion of TFBS of *SOC1* in the promoter of *AS1* is likely associated with late flowering time in wheat and *AS1* requires *SOC1* to induce flowering time in GA response. The direct interaction between *SOC1* and *AS1* has been not reported so far, even in the *Arabidopsis* background. Further explorations are necessary to confirm this interaction in vivo and in vitro*.*

## Conclusion

In the present study, we investigated the transcriptome profiling at the transition to the reproductive stage, which uncovered stage and spatial tissue-specific QTL in winter wheat. In total, 670 and 1075 DEGs in early “Kontrast” compared to late “Basalt” in SAM and leaves, respectively, could be mapped in 23 QTL intervals associated with heading time. We showed that the transition apex, double ridge stage, and reproductive phase are decisive steps in the floral switch process in which some key flowering time-related genes are activated for responding to external and internal stimuli such as light, ambient temperature, and day length change. The spatial expression of those genes in specific tissues grants first insights into possible cross-talk and signals migrations from leaves to the main shoot apex and vice versa. We have uncovered a potential antagonist of *VRN3* on chr 7D acting as a repressor of flowering time due to polymorphisms in critical amino acids of the coding sequence. The allele harboring SNPs are mapped in QTL177 showing significant association to heading trait. We detected the involvement of GA mechanisms in the flowering time pathway in wheat via the expression of *TraesCS5A02G079100* encoding *AS1* protein. *SOC1* binds in silico to a specific TFBS in the promoter of *AS1*, and both genes respond to GA biosynthesis for inducing flowering time. Our results enrich the knowledge and understanding gained so far in the transition to the reproductive phase in wheat on genetic and molecular levels.

## Supplementary Information

**Additional file 1: Fig. S1.** Mean vs. stability plot of heading date showing the principal components analysis of the stability/heterogeneity of 162 adapted cultivars bred in Germany.

**Additional file 2: Table S1.** List of significant QTL for heading in the Germany adapted cultivars.

**Additional file 3: Table S2.** List of primers used for PCR, RT-qPCR and sequencing of candidate genes.

**Additional file 4: Table S3.** Paired Student t-test of significant Waddington scores difference between cultivars.

**Additional file 5: Table S4**. List of the total annotated and mapped transcripts differentially expressed in SAM in 18 libraries.

**Additional file 6: Table S5**. List of the total annotated and mapped transcripts differentially expressed in leaves in 18 libraries.

**Additional file 7: Table S6.** Differential expressed genes in “Kontrast” compared to “Basalt” mapped in the QTL intervals in SAM.

**Additional file 8: Table S7.** Differential expressed genes in “Kontrast” compared to “Basalt” mapped in the QTL intervals in the leaves.

**Additional file 9: Table S8**. Gene ontology enrichment analysis in “Kontrast” relatively to “Basalt” in SAM.

**Additional file 10: Table S9**. Gene ontology enrichment analysis in “Kontrast” relatively to “Basalt” in the leaves.

**Additional file 11: Fig. S2.** qRT-PCR validation of the expression patterns of six randomly selected DEGs identified by RNA-seq in shoot apical meristem and leaves.

**Additional file 12: Fig. S3.** Alignment tree of the sequenced promoter region (2 kb upstream of the start codon) of *AS1* gene of the control and the cultivars “Kontrast” and “Basalt”.

**Additional file 13.** REVIGO TreeMap visualizations of significant GO terms

## Data Availability

RNA sequencing data have been deposited in the sequence read archive - SRA (https://www.ncbi.nlm.nih.gov/bioproject/PRJNA856668).
