# Supplementary material for: Transcriptome profiling at the transition to the reproductive stage uncovers stage and tissue-specific genes in wheat
Source: BMC Plant Biol. 2023 Jan 12;23:25. doi: 10.1186/s12870-022-03986-y (PMC9835304; doi:10.1186/s12870-022-03986-y)
Supplement: Supplementary file 1 — Additional file 1: Fig. S1. Mean vs. stability plot of heading date showing the principal components analysis of the stability/heterogeneity of 162 adapted cultivars bred in Germany. [file 12870_2022_3986_MOESM1_ESM.docx]

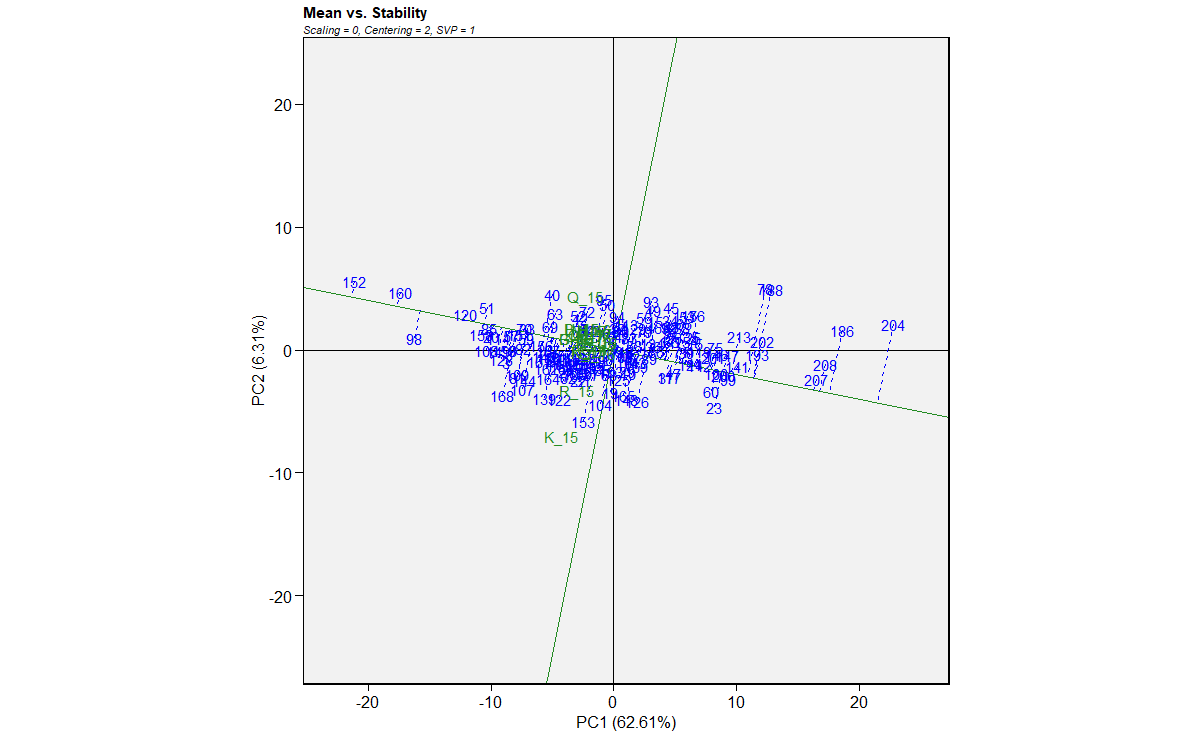


Additional file 1: Mean vs. stability plot of heading date showing the principal components analysis of the stability/heterogeneity of 162 adapted cultivars bred in Germany. The early flowering cultivar 207 “Kontrast “and the late flowering one 152 “Basalt” are selected for their stable flowering behavior in different environments indicated in green (six locations and three years, Benaouda et al., 2022), cultivars are shown in blue. The green line passing through the biplot is referring to the average-environmental axis. The early flowering cultivars are clustered on the right side of the plot, the late flowering ones on the left side. The closest the cultivar to the green line, the more stable in all environments.
