# Supplementary material for: Transcriptome profiling at the transition to the reproductive stage uncovers stage and tissue-specific genes in wheat
Source: BMC Plant Biol. 2023 Jan 12;23:25. doi: 10.1186/s12870-022-03986-y (PMC9835304; doi:10.1186/s12870-022-03986-y)
Supplement: Supplementary file 11 — Additional file 11: Fig. S2. qRT-PCR validation of the expression patterns of six randomly selected DEGs identified by RNA-seq in shoot apical meristem and leaves. [file 12870_2022_3986_MOESM11_ESM.docx]

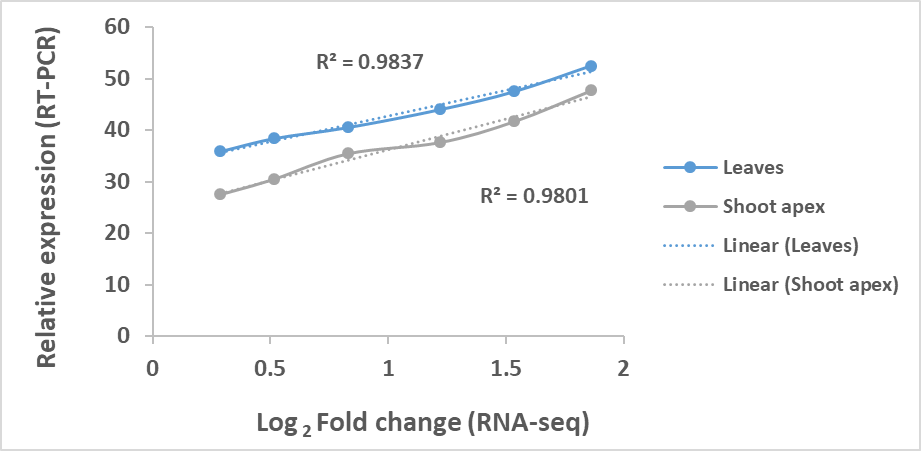


Additional file 11: qRT-PCR validation of the expression patterns of six randomly selected DEGs identified by RNA-seq in shoot apical meristem and leaves.
