# Supplementary material for: Transcriptome profiling at the transition to the reproductive stage uncovers stage and tissue-specific genes in wheat
Source: BMC Plant Biol. 2023 Jan 12;23:25. doi: 10.1186/s12870-022-03986-y (PMC9835304; doi:10.1186/s12870-022-03986-y)
Supplement: Supplementary file 12 — Additional file 12: Fig. S3. Alignment tree of the sequenced promoter region (2 kb upstream of the start codon) of AS1 gene of the control and the cultivars “Kontrast” and “Basalt”. [file 12870_2022_3986_MOESM12_ESM.docx]

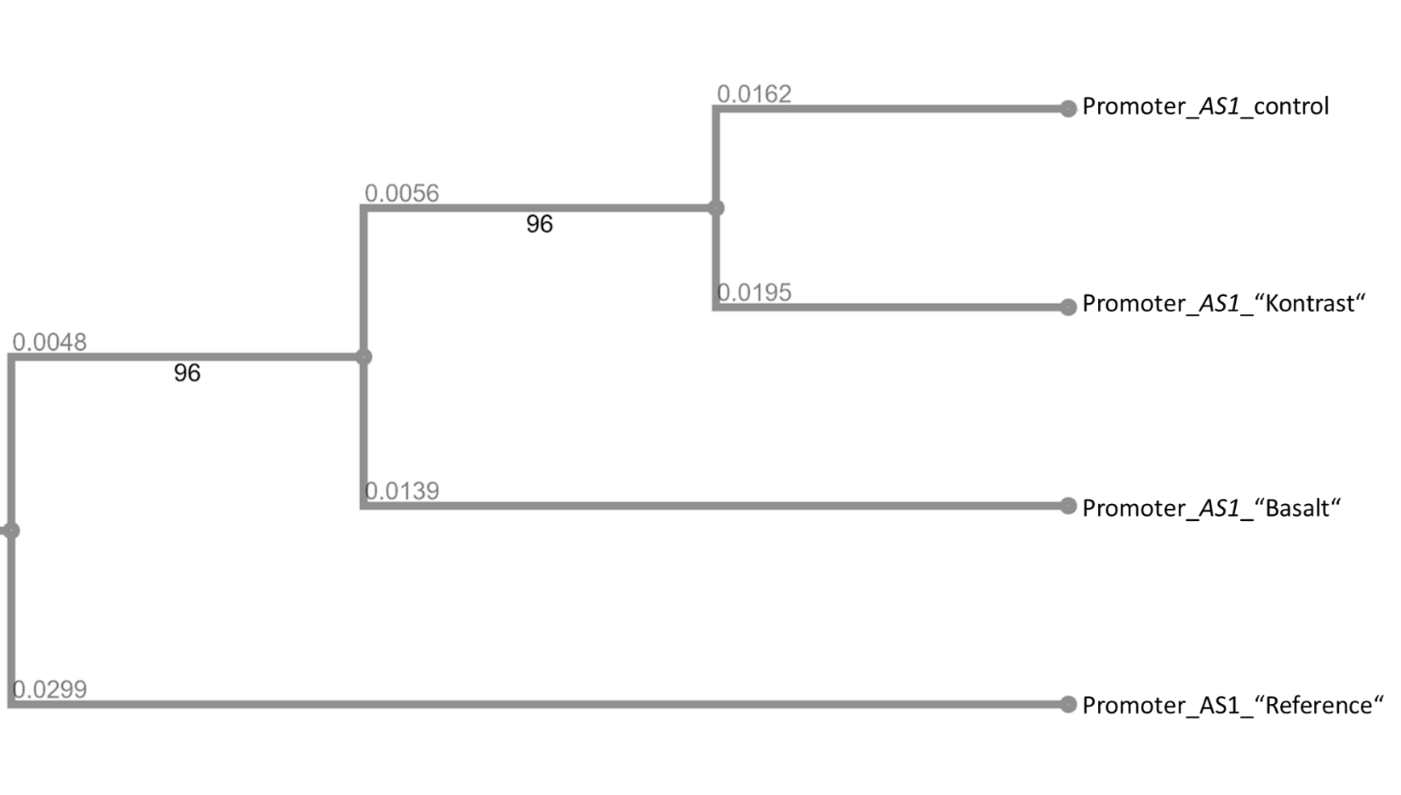


Additional file 12: Alignment tree of the sequenced promoter region (2kb upstream of the start codon) of *AS1* gene of the control and the cultivars “Kontrast” and “Basalt”. Level of significance is indicated in grey numbers. Percentage of the shared sequence is highlighted in black.
