# Supplementary material for: Transcriptome profiling at the transition to the reproductive stage uncovers stage and tissue-specific genes in wheat
Source: BMC Plant Biol. 2023 Jan 12;23:25. doi: 10.1186/s12870-022-03986-y (PMC9835304; doi:10.1186/s12870-022-03986-y)

Additional file 13: “TreeMap” views of significant GO terms constructed with REVIGO (Supek et al., 2011). Each rectangle is a single cluster representative. The representatives are joined into ‘superclusters’ of loosely related terms, visualized with different colors. Size of the rectangles reflect the *p*-value.

Supek, Fran, et al. "REVIGO summarizes and visualizes long lists of gene ontology terms." PloS one 6.7 (2011): e21800.

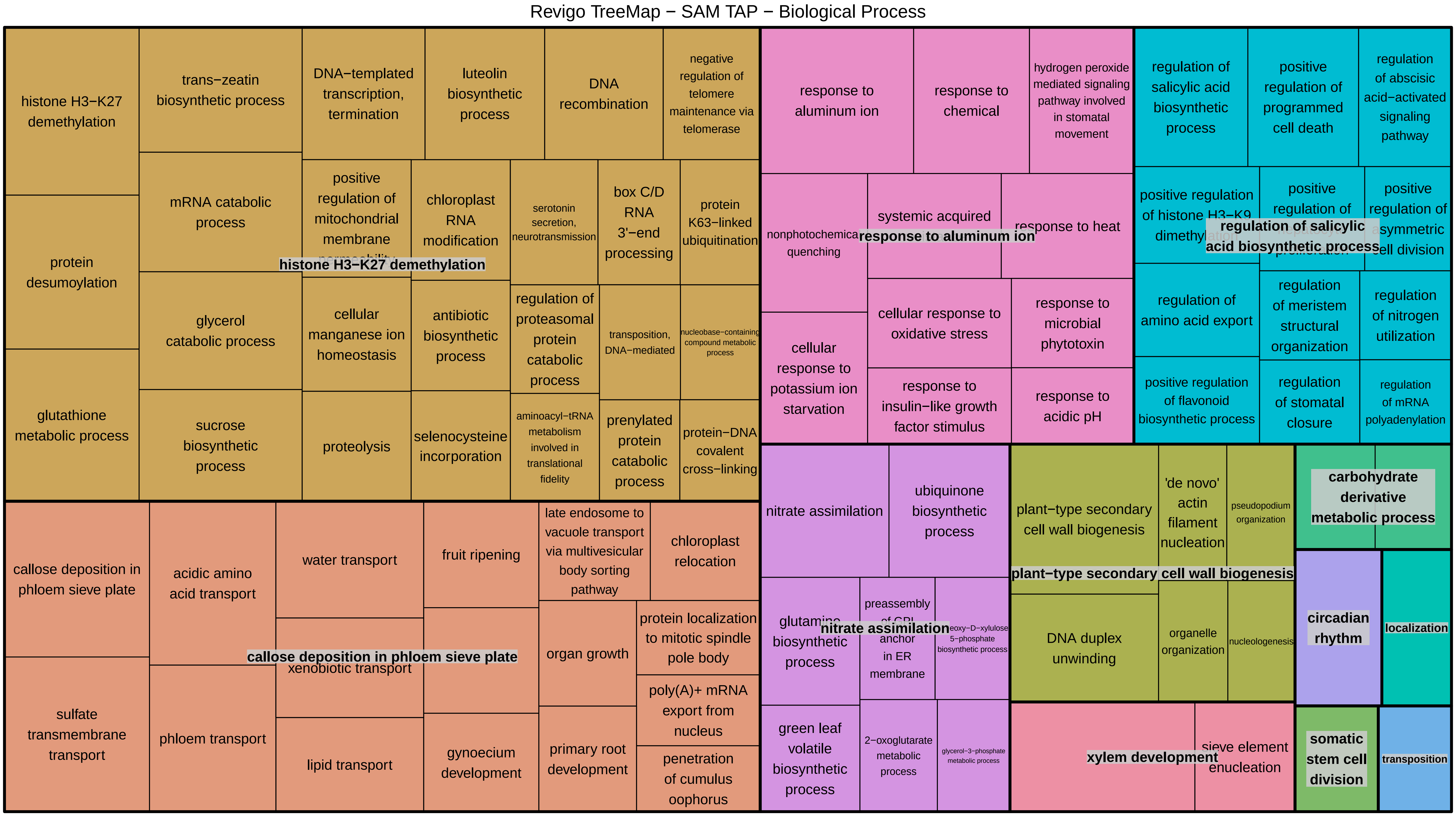
Revigo TreeMap − SAM TAP − Biological Process
histone H3−K27 demethylation
trans−zeatin biosynthetic process
DNA−templated transcription, termination
luteolin biosynthetic process
DNA recombination
negative regulation of telomere maintenance via telomerase
protein desumoylation
mRNA catabolic process
positive regulation of mitochondrial membrane permeability
chloroplast RNA modification
serotonin secretion, neurotransmission
box C/D RNA 3'−end processing
protein K63−linked ubiquitination
glutathione metabolic process
glycerol catabolic process
cellular manganese ion homeostasis
antibiotic biosynthetic process
regulation of proteasomal protein catabolic process
transposition, DNA−mediated
nucleobase−containing compound metabolic process
sucrose biosynthetic process
proteolysis
selenocysteine incorporation
aminoacyl−tRNA metabolism involved in translational fidelity
prenylated protein catabolic process
protein−DNA covalent cross−linking
response to aluminum ion
response to chemical
hydrogen peroxide mediated signaling pathway involved in stomatal movement
nonphotochemical quenching
systemic acquired
response to heat
cellular response to potassium ion starvation
cellular response to oxidative stress
response to microbial phytotoxin
response to insulin−like growth factor stimulus
response to acidic pH
regulation of salicylic acid biosynthetic process
positive regulation of programmed cell death
regulation of abscisic acid−activated signaling pathway
positive regulation of histone H3−K9 dimethylation
positive regulation of
positive regulation of asymmetric cell division
regulation of amino acid export
regulation of meristem structural organization
regulation of nitrogen utilization
positive regulation of flavonoid biosynthetic process
regulation of stomatal closure
regulation of mRNA polyadenylation
callose deposition in phloem sieve plate
acidic amino acid transport
water transport
fruit ripening
late endosome to vacuole transport via multivesicular body sorting pathway
chloroplast relocation
xenobiotic transport
organ growth
protein localization to mitotic spindle pole body
sulfate transmembrane transport
phloem transport
lipid transport
gynoecium development
primary root development
poly(A)+ mRNA export from nucleus
penetration of cumulus oophorus
nitrate assimilation
ubiquinone biosynthetic process
glutamine biosynthetic process
preassembly of GPI anchor in ER membrane
deoxy−D−xylulose 5−phosphate biosynthetic process
green leaf volatile biosynthetic process
2−oxoglutarate metabolic process
glycerol−3−phosphate metabolic process
plant−type secondary cell wall biogenesis
'de novo' actin filament nucleation
pseudopodium organization
DNA duplex unwinding
organelle organization
nucleologenesis
xylem development
sieve element enucleation
carbohydrate derivative metabolic process
circadian rhythm
localization
somatic stem cell division
transposition

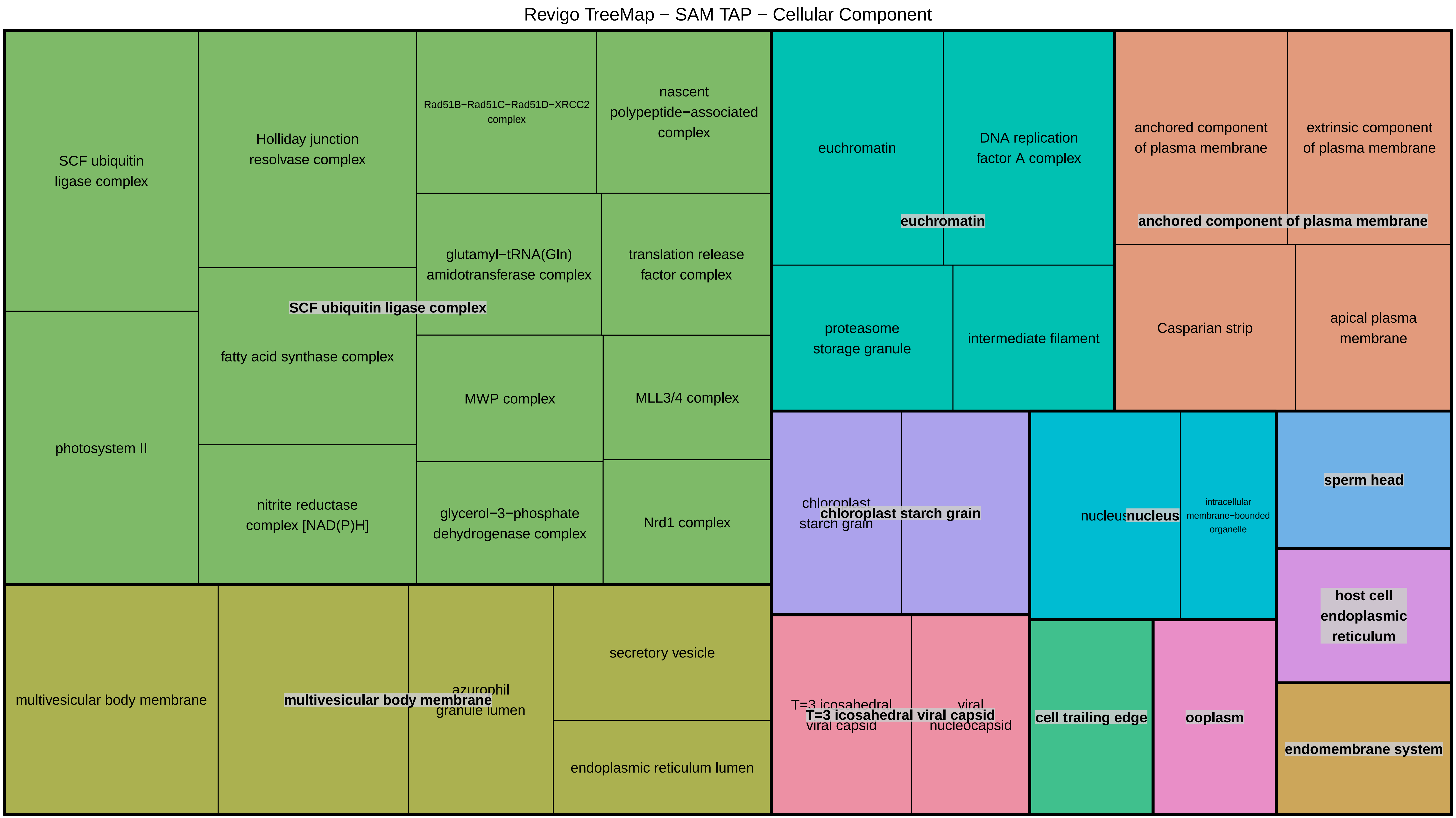
Revigo TreeMap − SAM TAP − Cellular Component
SCF ubiquitin ligase complex
Holliday junction resolvase complex
Rad51B−Rad51C−Rad51D−XRCC2 complex
nascent polypeptide−associated complex
glutamyl−tRNA(Gln) amidotransferase complex
translation release factor complex
SCF ubiquitin ligase complex
fatty acid synthase complex
MWP complex
MLL3/4 complex
photosystem II
nitrite reductase complex [NAD(P)H]
glycerol−3−phosphate dehydrogenase complex
Nrd1 complex
multivesicular body membrane
multivesicular body membrane
azurophil granule lumen
secretory vesicle
endoplasmic reticulum lumen
euchromatin
DNA replication factor A complex
euchromatin
proteasome storage granule
intermediate filament
anchored component of plasma membrane
extrinsic component of plasma membrane
anchored component of plasma membrane
Casparian strip
apical plasma membrane
chloroplast starch grain
chloroplast starch grain
nucleus
nucleus
intracellular membrane−bounded organelle
sperm head
host cell endoplasmic reticulum
T=3 icosahedral viral capsid
T=3 icosahedral viral capsid
viral nucleocapsid
cell trailing edge
ooplasm
endomembrane system

Revigo TreeMap − SAM TAP − Molecular Function

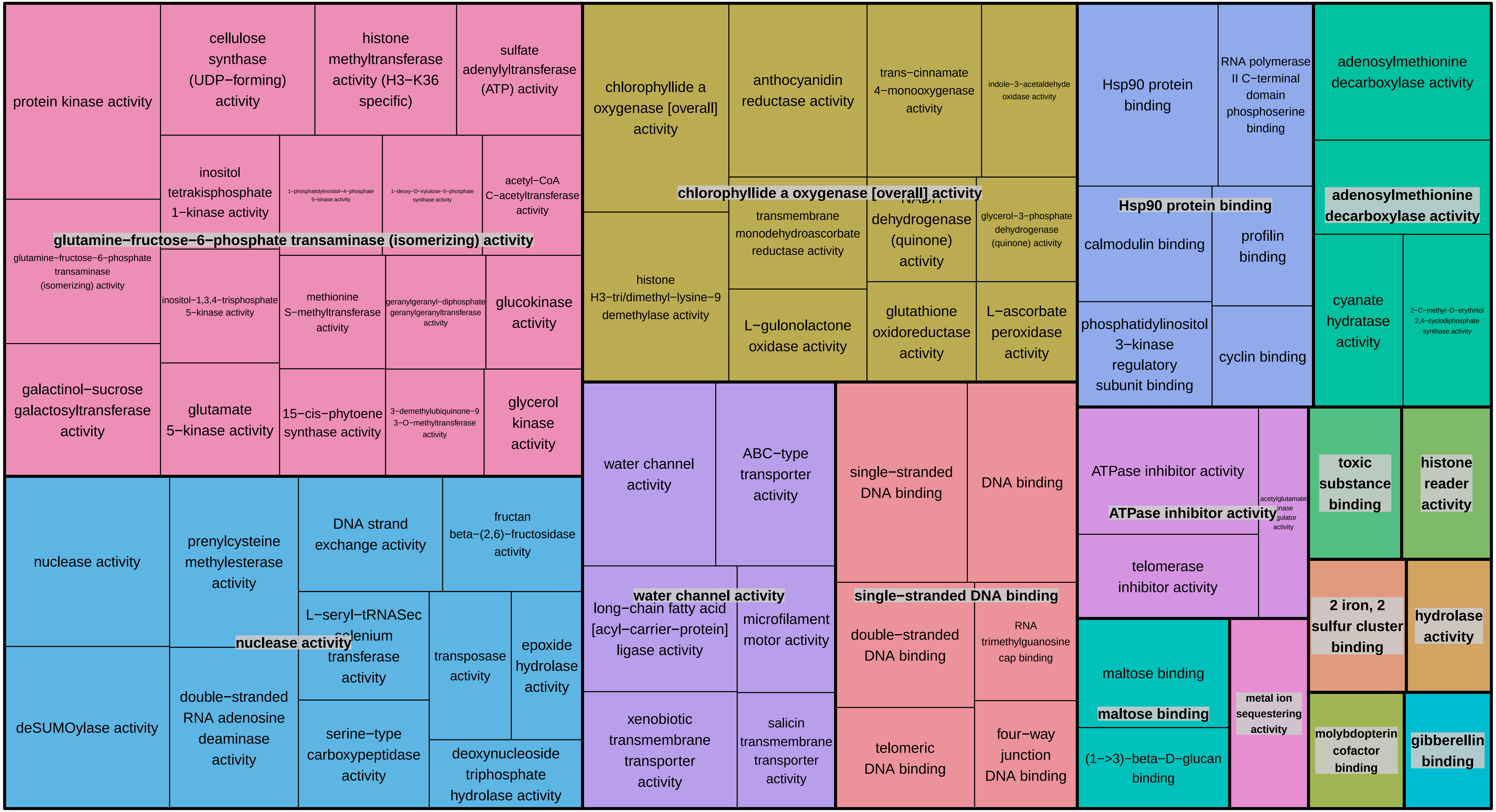

Revigo TreeMap − SAM DRS − Biological Process

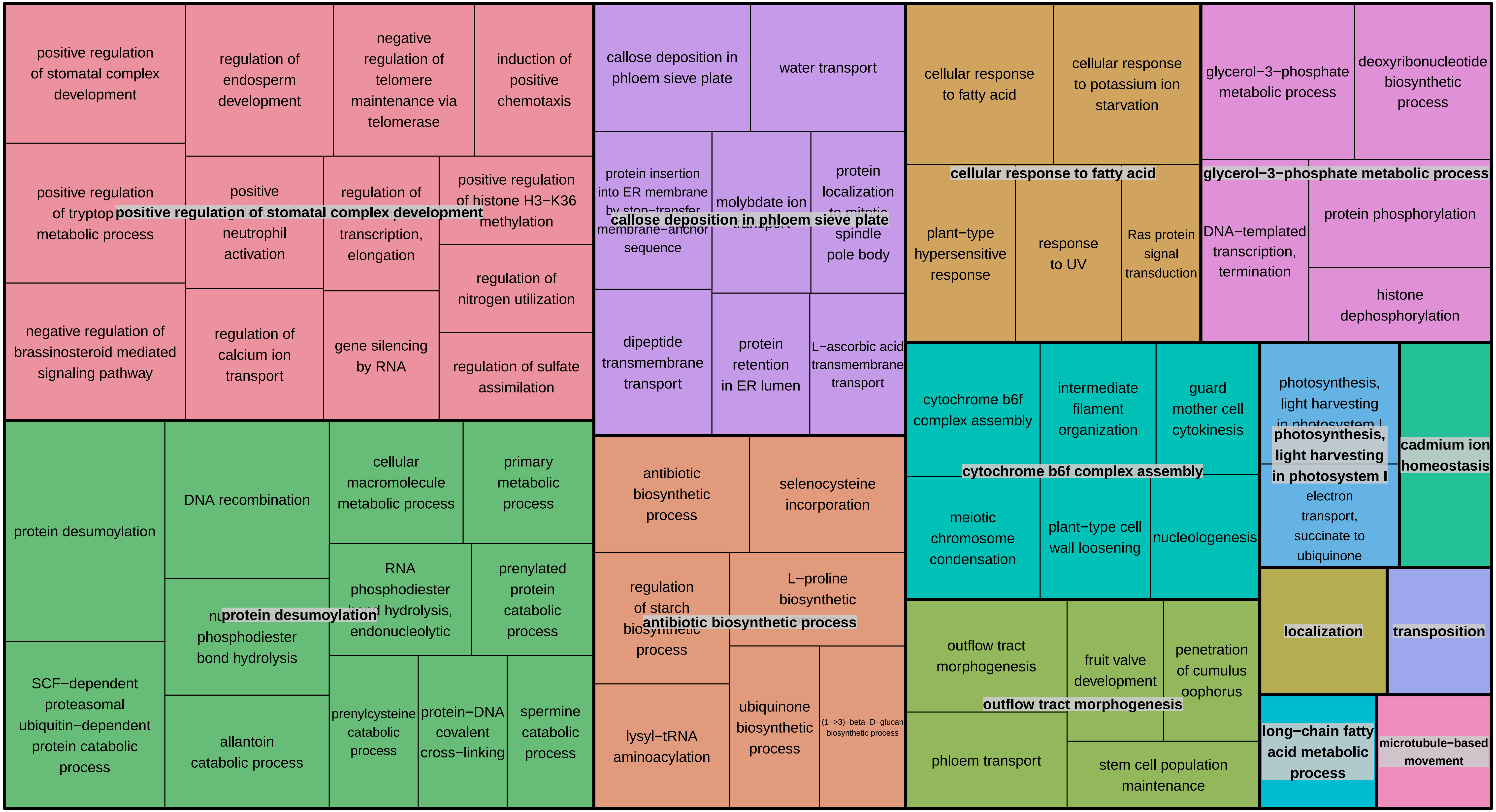

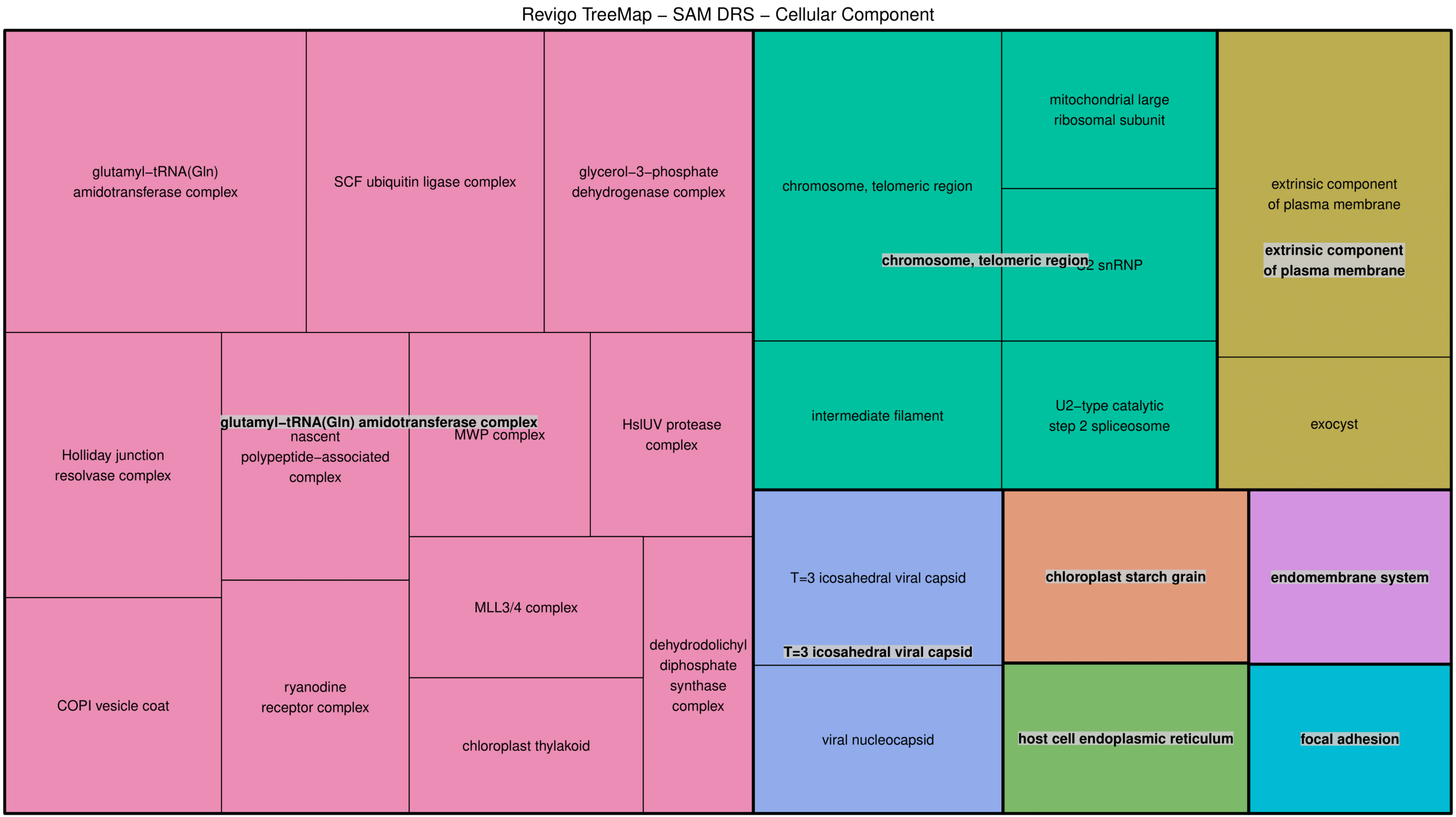
Revigo TreeMap – SAM DRS – Cellular Component
glutamyl–tRNA(Gln) amidotransferase complex
SCF ubiquitin ligase complex
glycerol–3–phosphate dehydrogenase complex
Holliday junction resolvase complex
glutamyl–tRNA(Gln) amidotransferase complex
nascent polypeptide–associated complex
MWP complex
HslUV protease complex
COPI vesicle coat
ryanodine receptor complex
MLL3/4 complex
chloroplast thylakoid
dehydrodolichyl diphosphate synthase complex
chromosome, telomeric region
mitochondrial large ribosomal subunit
chromosome, telomeric region
U2 snRNP
intermediate filament
U2–type catalytic step 2 spliceosome
extrinsic component of plasma membrane
extrinsic component of plasma membrane
exocyst
T=3 icosahedral viral capsid
T=3 icosahedral viral capsid
viral nucleocapsid
chloroplast starch grain
endomembrane system
host cell endoplasmic reticulum
focal adhesion

Revigo TreeMap − SAM DRS − Molecular Function

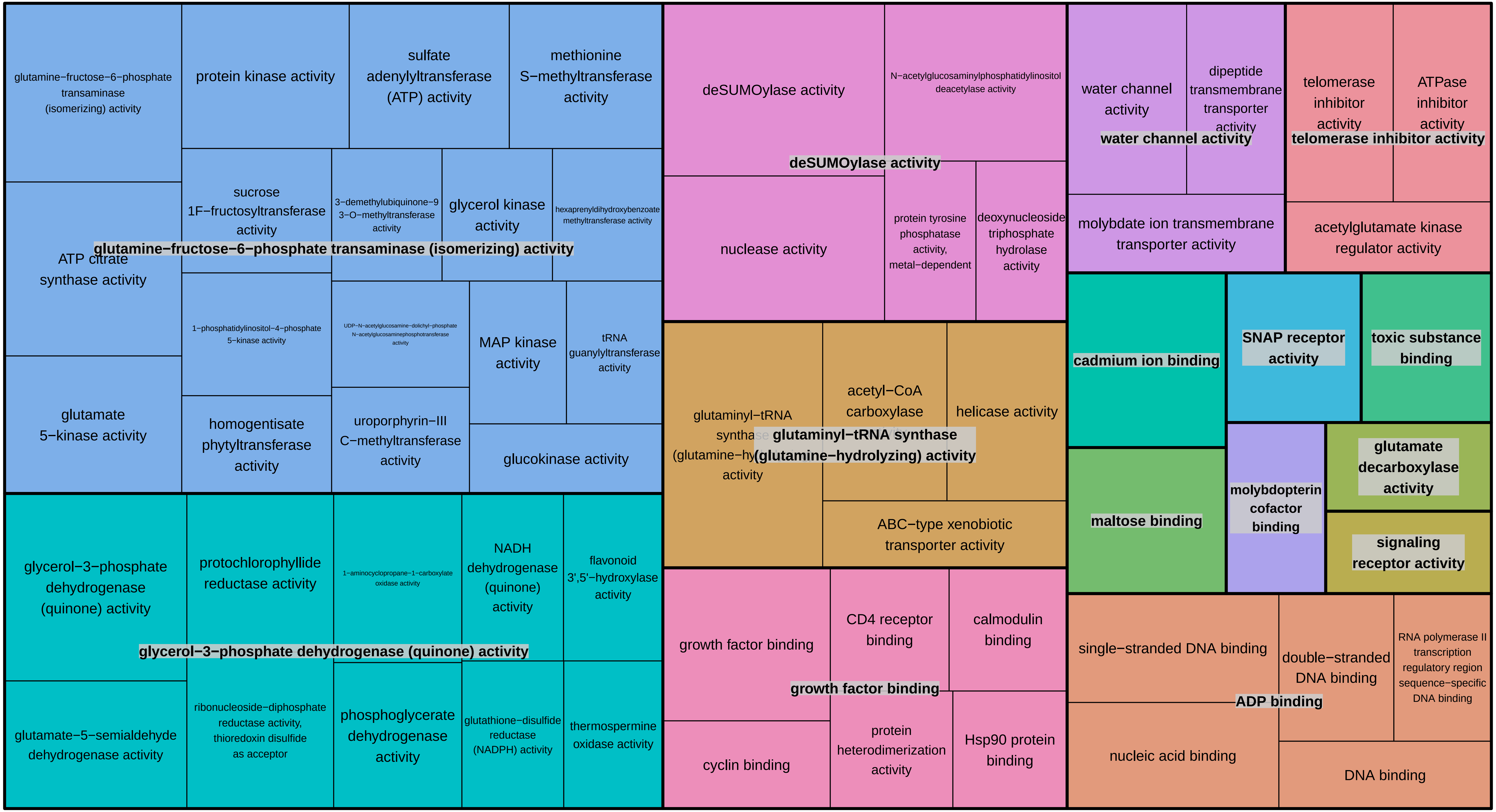

Revigo TreeMap − SAM LRP − Biological Process

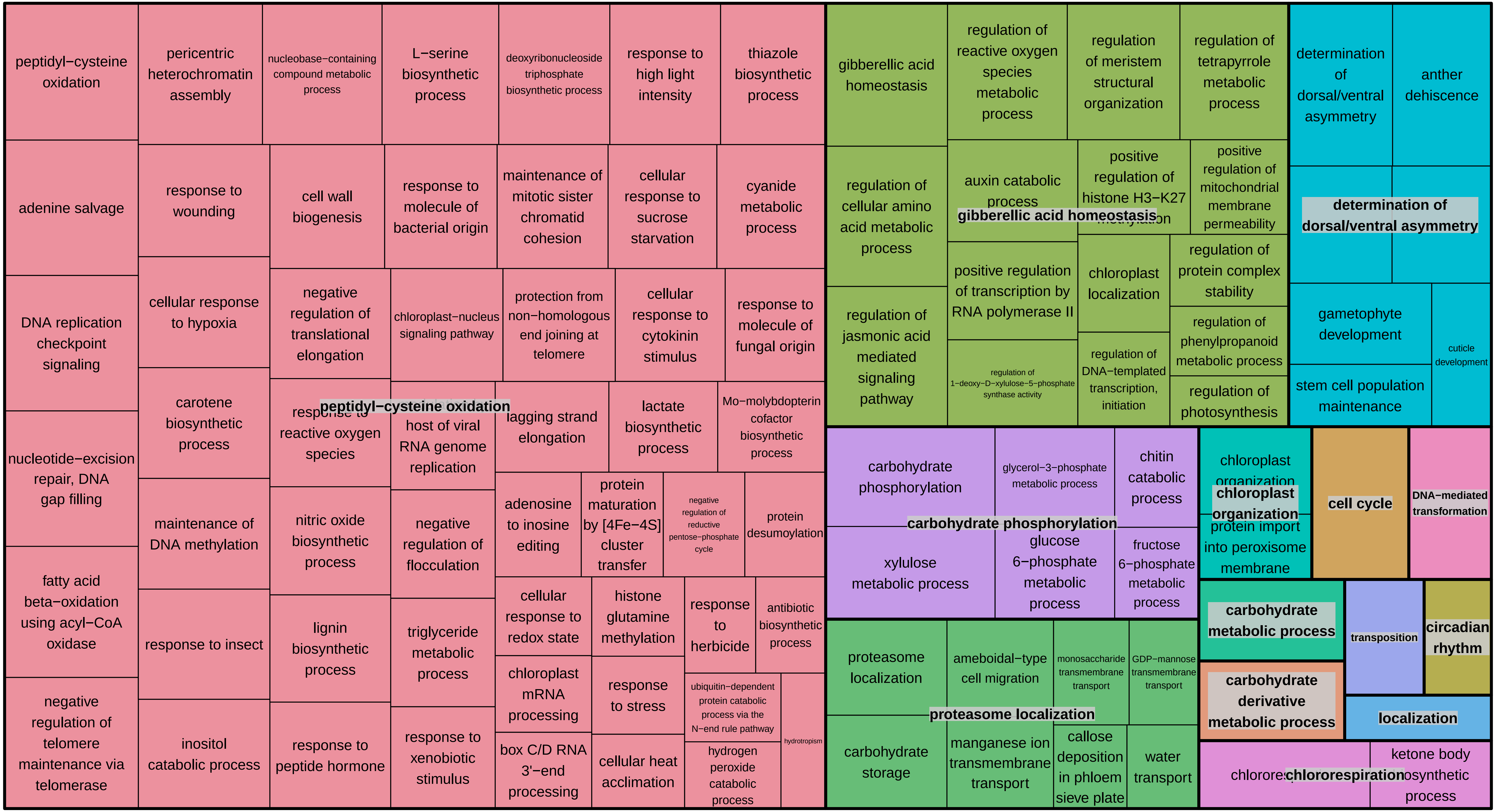

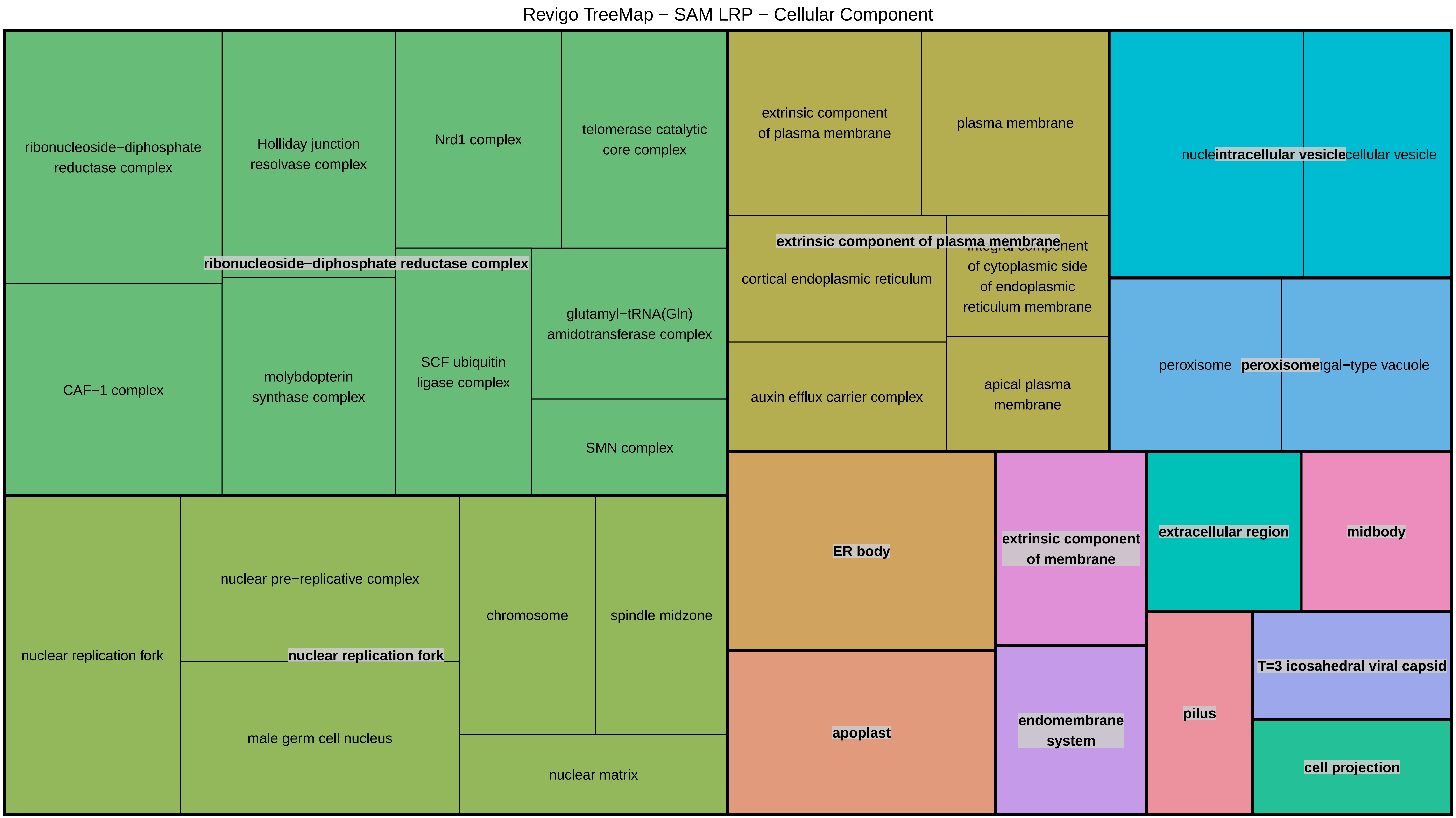

Revigo TreeMap − SAM LRP − Cellular Component
ribonucleoside−diphosphate reductase complex
Holliday junction resolvase complex
Nrd1 complex
telomerase catalytic core complex
ribonucleoside−diphosphate reductase complex
CAF−1 complex
molybdopterin synthase complex
SCF ubiquitin ligase complex
glutamyl−tRNA(Gln) amidotransferase complex
SMN complex
extrinsic component of plasma membrane
plasma membrane
extrinsic component of plasma membrane
cortical endoplasmic reticulum
integral component of cytoplasmic side of endoplasmic reticulum membrane
auxin efflux carrier complex
apical plasma membrane
intracellular vesicle
peroxisome
nuclear replication fork
nuclear pre−replicative complex
nuclear replication fork
male germ cell nucleus
chromosome
spindle midzone
nuclear matrix
ER body
apoplast
extrinsic component of membrane
endomembrane system
extracellular region
midbody
pilus
T=3 icosahedral viral capsid
cell projection

Revigo TreeMap − SAM LRP − Molecular Function

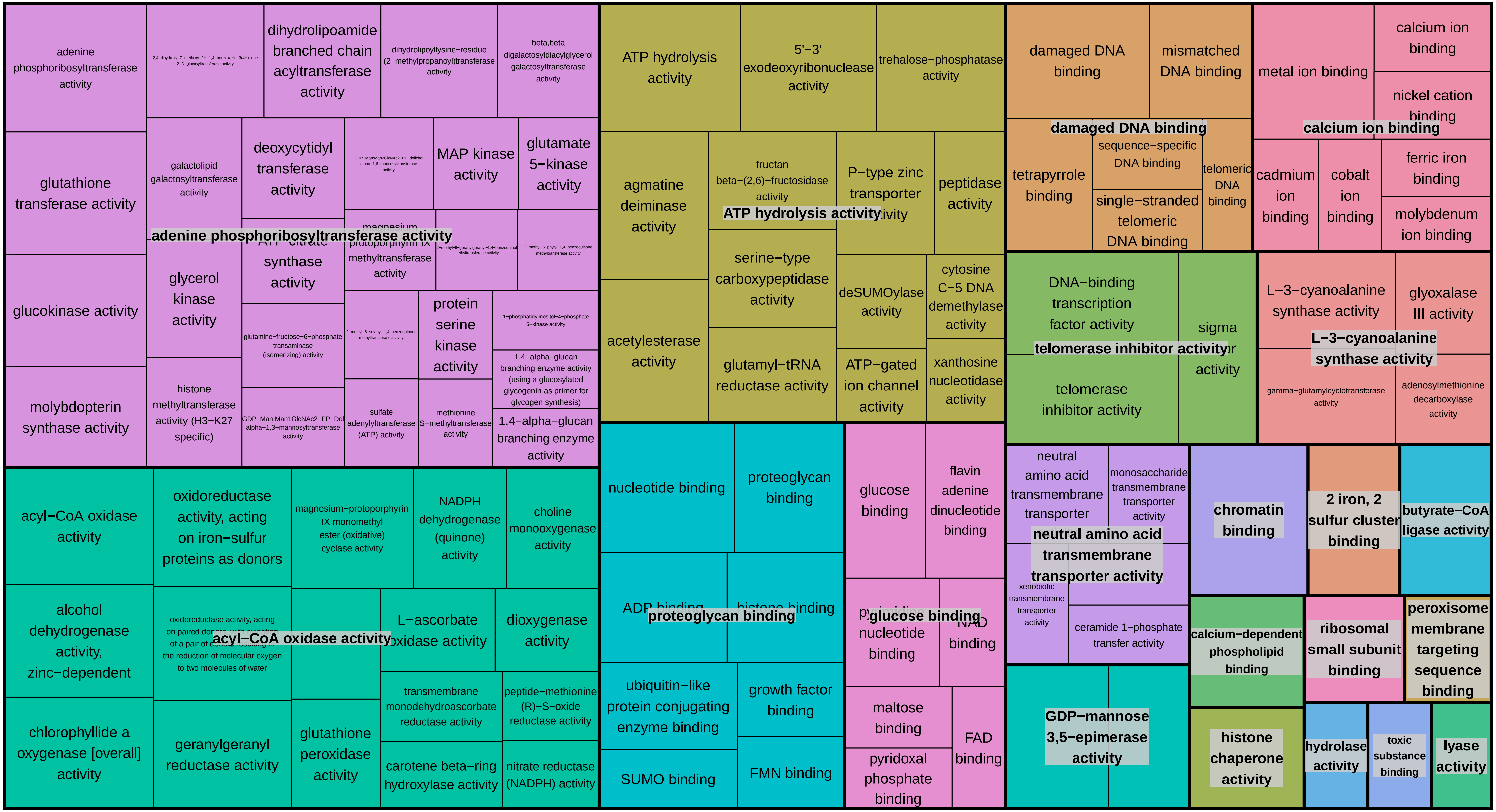

Revigo TreeMap − Leaves Tap − Biological Process

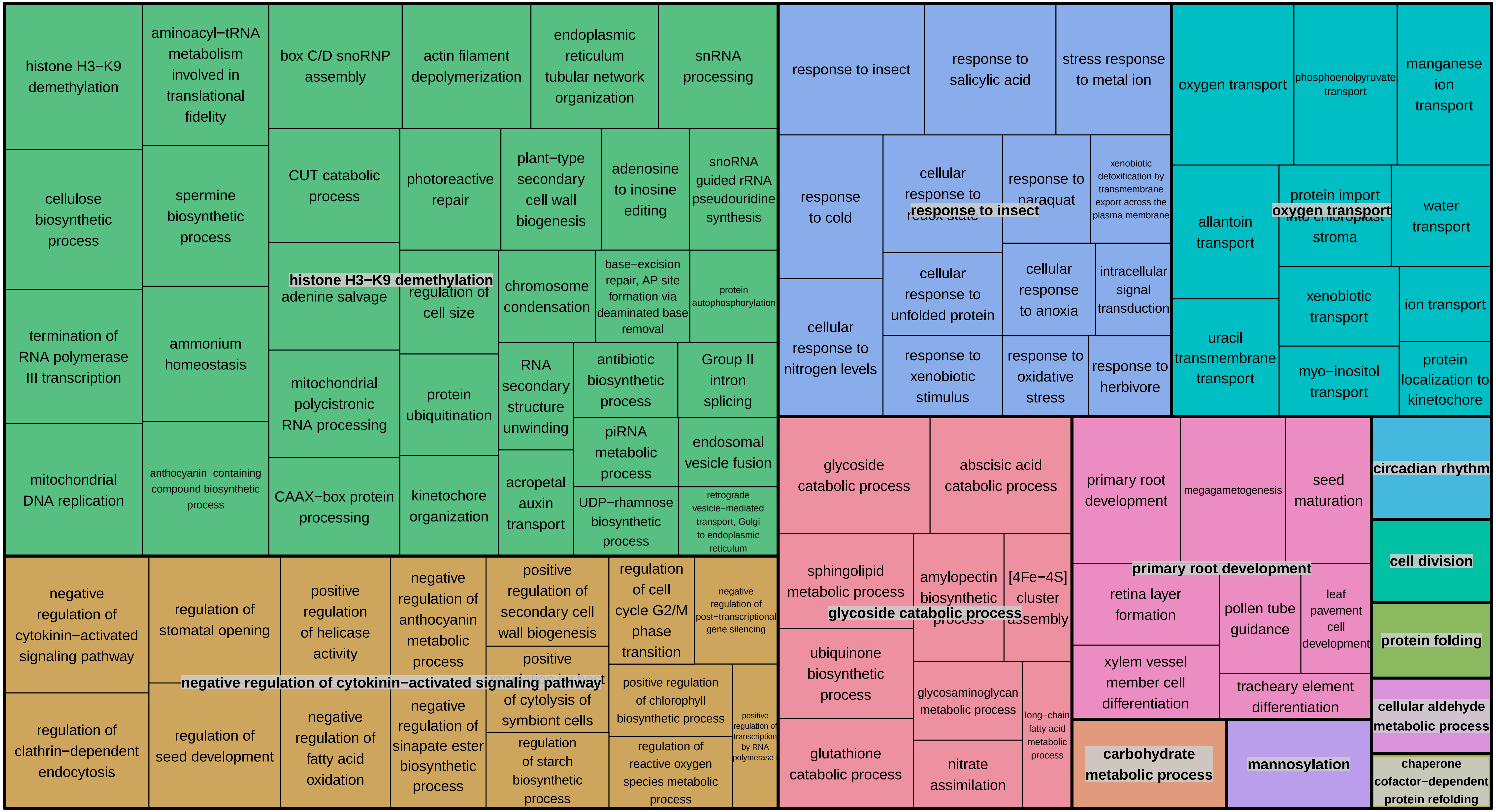

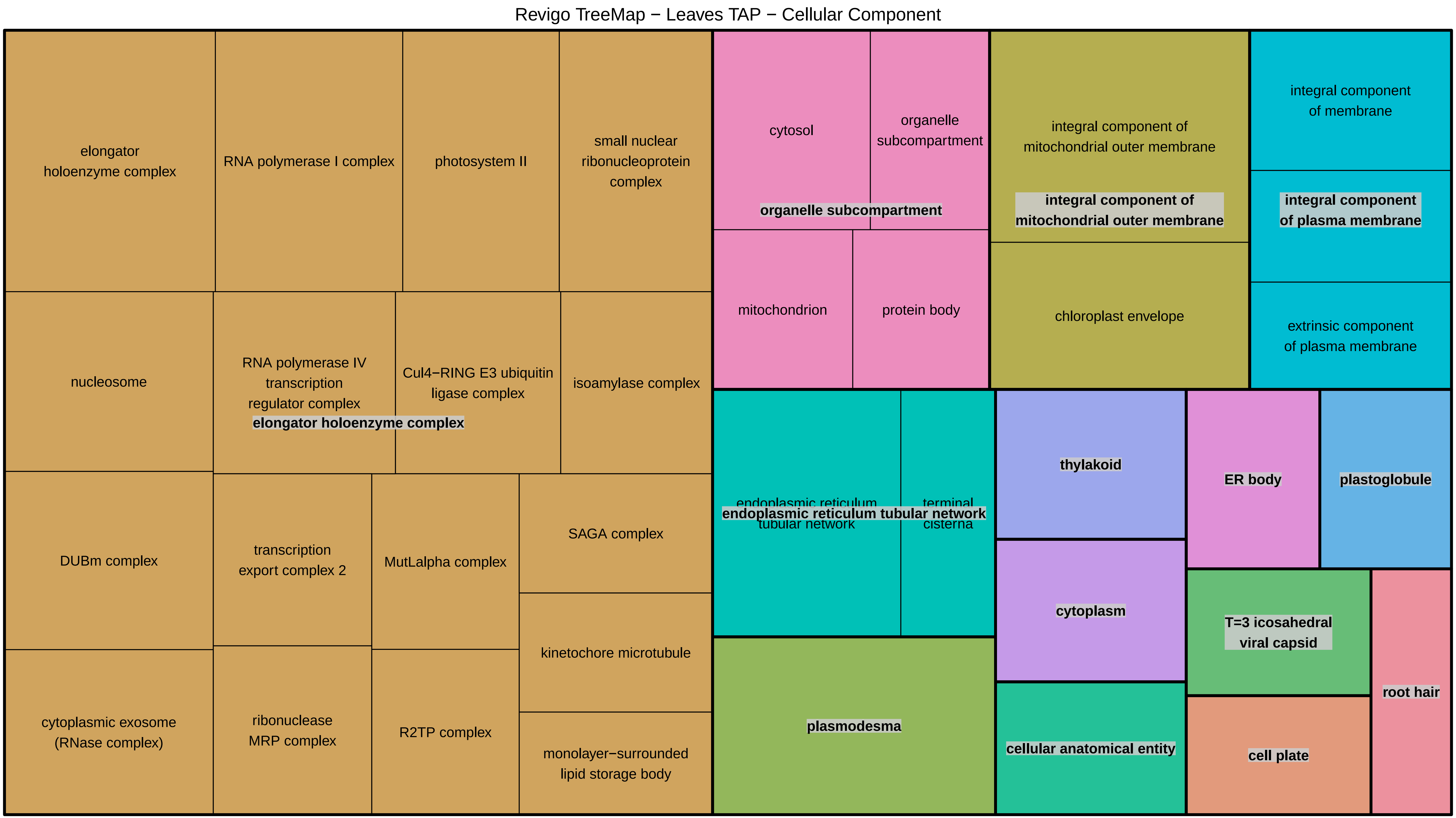
Revigo TreeMap − Leaves TAP − Cellular Component
elongator holoenzyme complex
RNA polymerase I complex
photosystem II
small nuclear ribonucleoprotein complex
nucleosome
RNA polymerase IV transcription regulator complex
Cul4−RING E3 ubiquitin ligase complex
isoamylase complex
elongator holoenzyme complex
DUBm complex
transcription export complex 2
MutLalpha complex
SAGA complex
kinetochore microtubule
cytoplasmic exosome (RNase complex)
ribonuclease MRP complex
R2TP complex
monolayer−surrounded lipid storage body
cytosol
organelle subcompartment
organelle subcompartment
mitochondrion
protein body
integral component of mitochondrial outer membrane
integral component of mitochondrial outer membrane
chloroplast envelope
integral component of membrane
integral component of plasma membrane
extrinsic component of plasma membrane
endoplasmic reticulum tubular network
terminal cisterna
endoplasmic reticulum tubular network
plasmodesma
thylakoid
cytoplasm
cellular anatomical entity
ER body
plastoglobule
T=3 icosahedral viral capsid
cell plate
root hair

Revigo TreeMap − Leaves TAP − Molecular Function

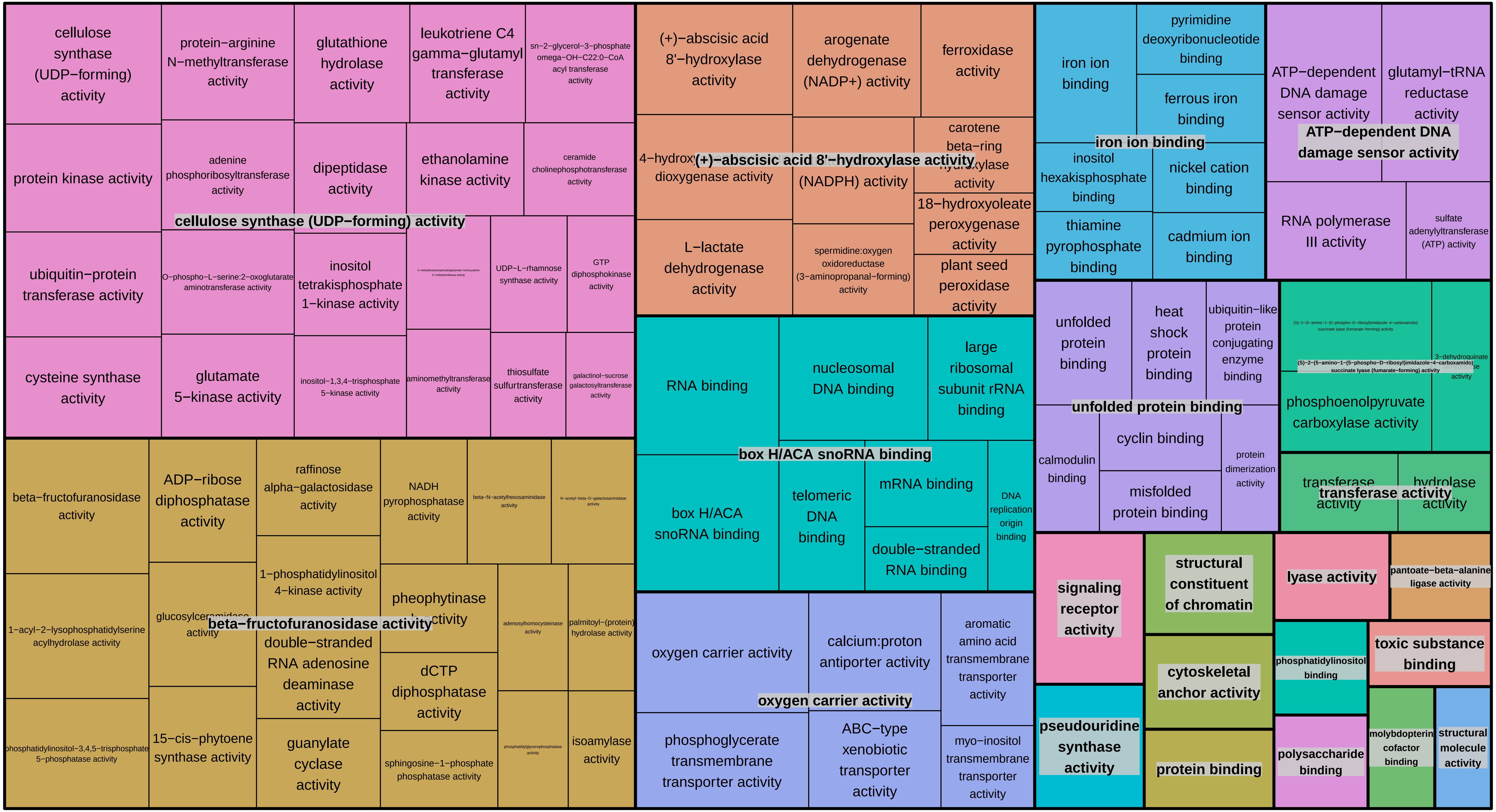

Revigo TreeMap − Leaves DRS − Biological Process

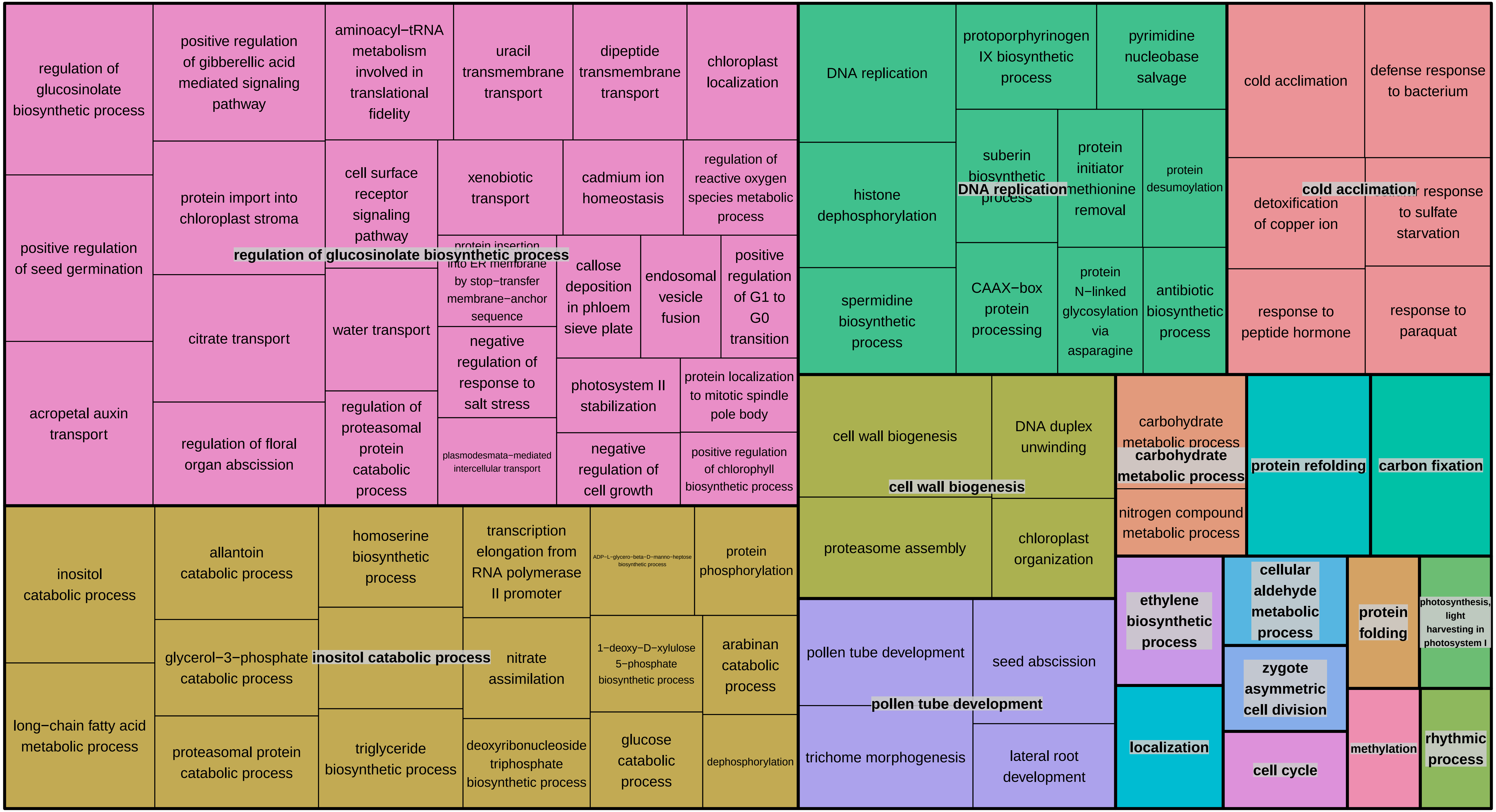

Revigo TreeMap − Leaves DRS − Cellular Component

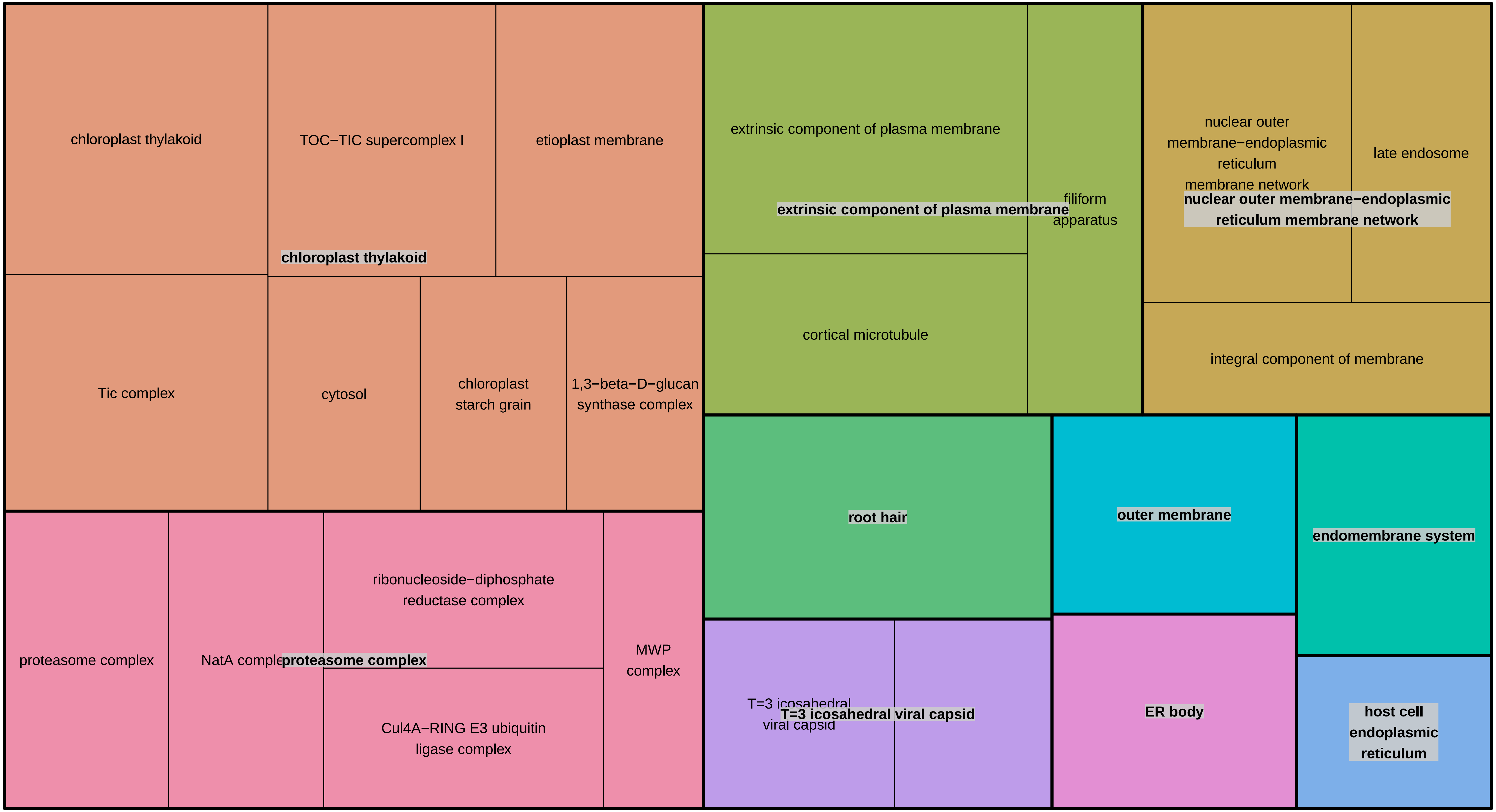

Revigo TreeMap − Leaves DRS − Molecular Function

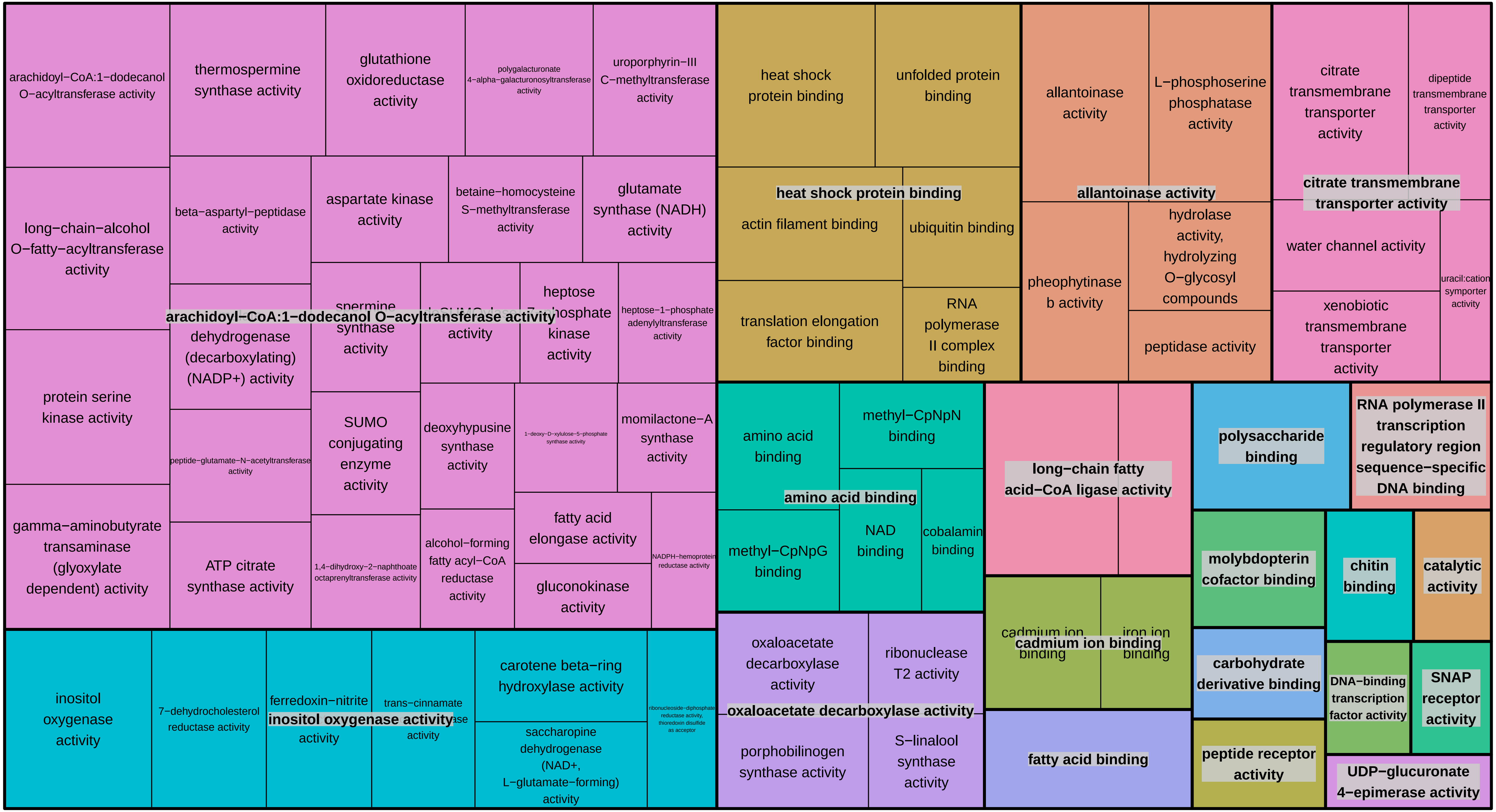

Revigo TreeMap − Leaves LRP − Biological Process

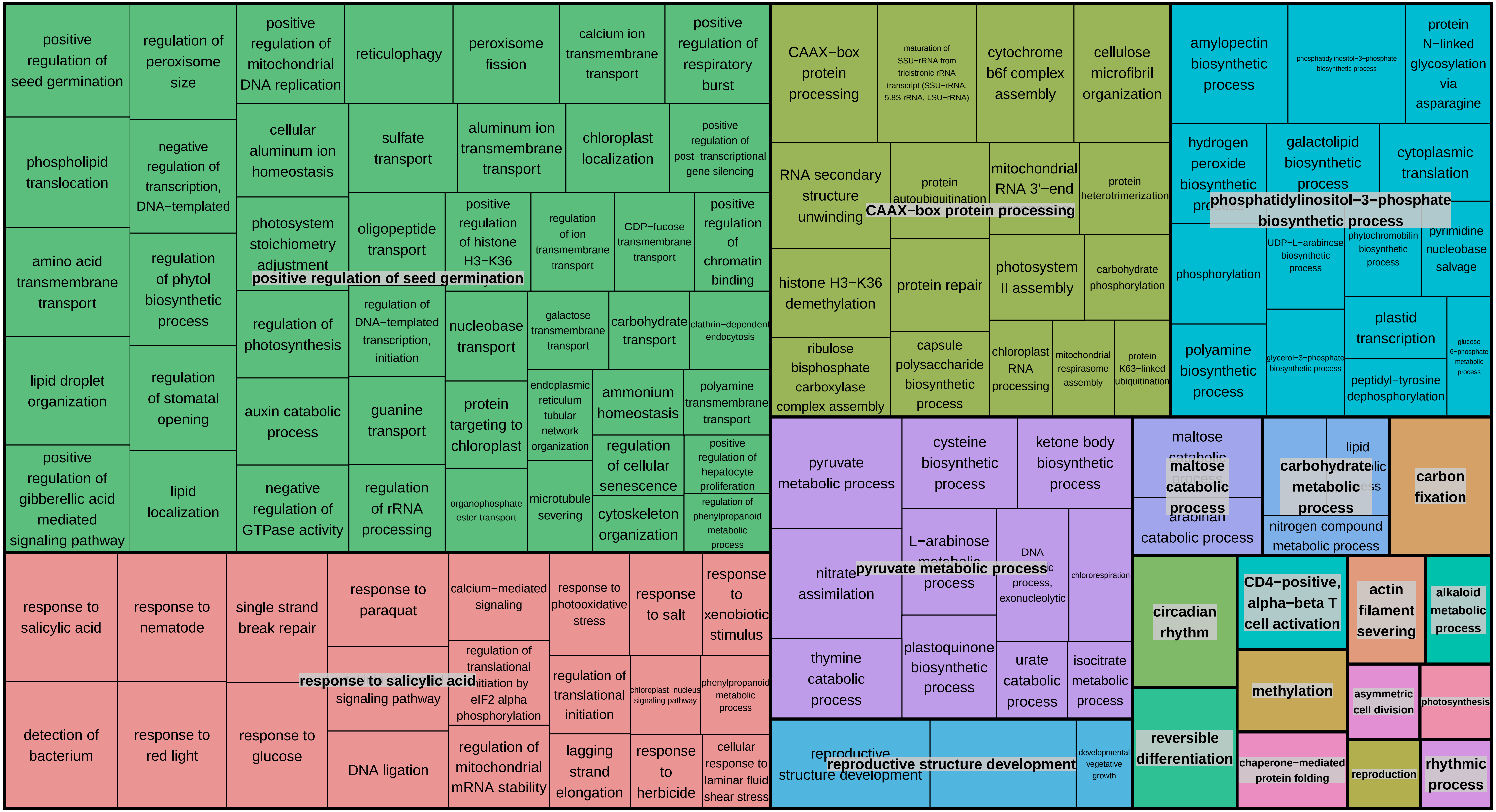

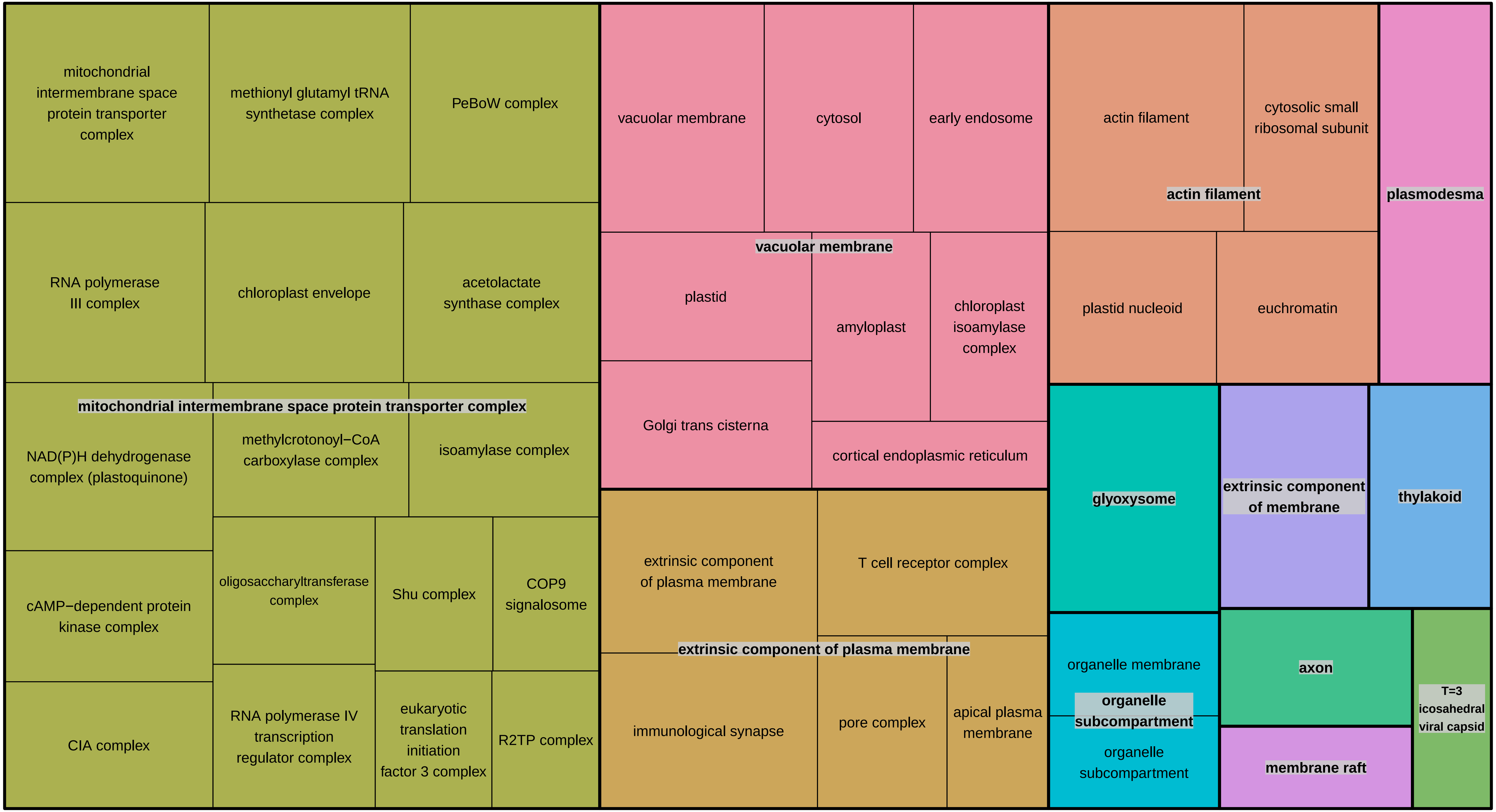
Revigo TreeMap − Leaves LRP − Cellular Component
mitochondrial intermembrane space protein transporter complex
methionyl glutamyl tRNA synthetase complex
PeBoW complex
RNA polymerase III complex
chloroplast envelope
acetolactate synthase complex
mitochondrial intermembrane space protein transporter complex
NAD(P)H dehydrogenase complex (plastoquinone)
methylcrotonoyl−CoA carboxylase complex
isoamylase complex
cAMP−dependent protein kinase complex
oligosaccharyltransferase complex
Shu complex
COP9 signalosome
CIA complex
RNA polymerase IV transcription regulator complex
eukaryotic translation initiation factor 3 complex
R2TP complex
vacuolar membrane
cytosol
early endosome
vacuolar membrane
plastid
amyloplast
chloroplast isoamylase complex
Golgi trans cisterna
cortical endoplasmic reticulum
extrinsic component of plasma membrane
T cell receptor complex
extrinsic component of plasma membrane
immunological synapse
pore complex
apical plasma membrane
actin filament
cytosolic small ribosomal subunit
actin filament
plastid nucleoid
euchromatin
plasmodesma
glyoxysome
extrinsic component of membrane
thylakoid
organelle membrane
organelle subcompartment
organelle subcompartment
axon
membrane raft
T=3 icosahedral viral capsid

Revigo TreeMap − Leaves LRP − Molecular Function

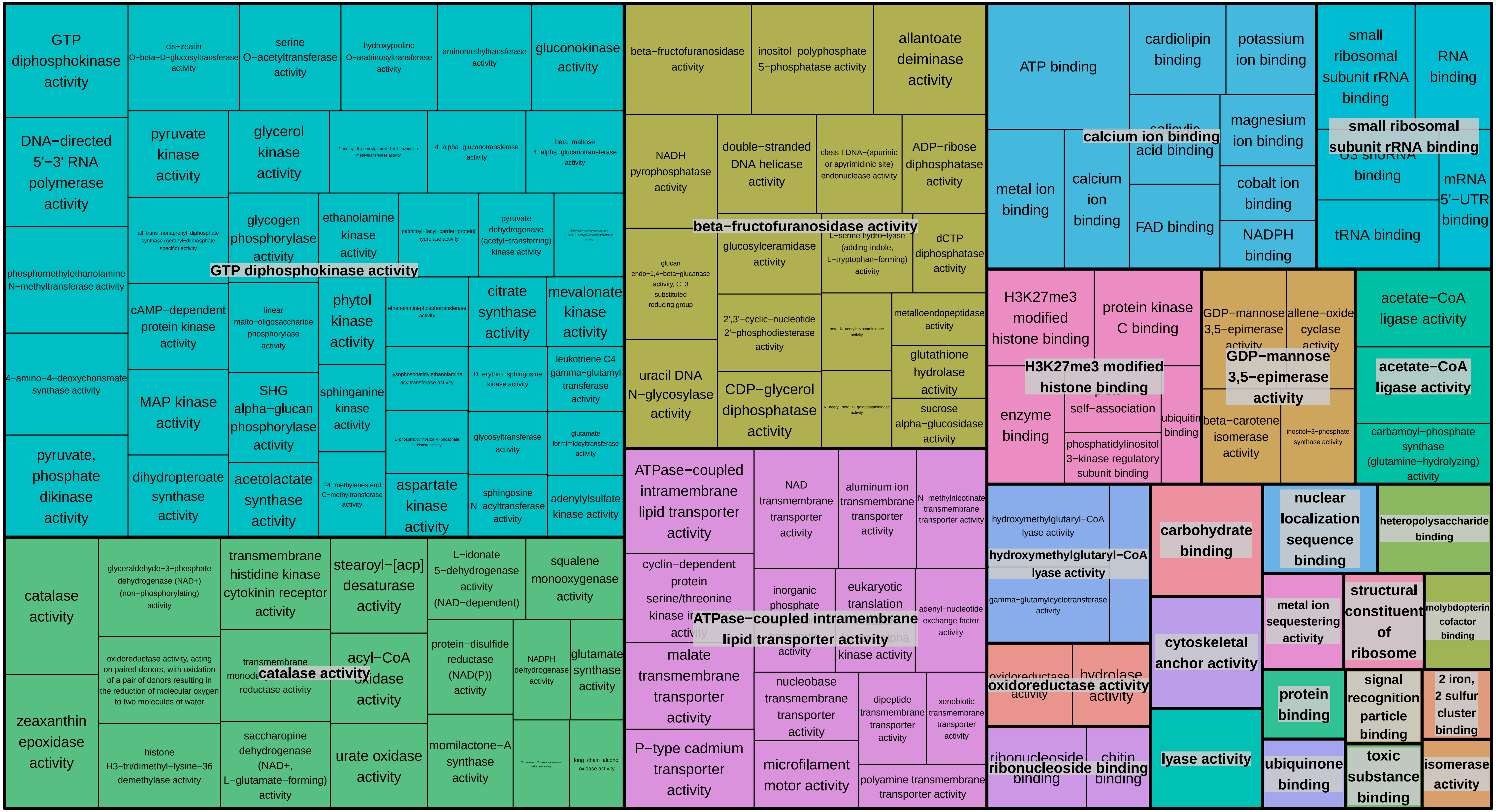

Supplement: Supplementary file 13 — Additional file 13. REVIGO TreeMap visualizations of significant GO terms [file 12870_2022_3986_MOESM13_ESM.pdf]
